# Hypercholesterolemia Is Associated with the Apolipoprotein C-III (*APOC3*) Genotype in Children Receiving HAART: An Eight-Year Retrospective Study

**DOI:** 10.1371/journal.pone.0039678

**Published:** 2012-07-25

**Authors:** Carlos A. Rocco, Debora Mecikovsky, Paula Aulicino, Rosa Bologna, Luisa Sen, Andrea Mangano

**Affiliations:** 1 Laboratorio de Biología Celular y Retrovirus, Hospital de Pediatría “Juan P. Garrahan”, Buenos Aires, Argentina; 2 Servicio de Epidemiología e Infectología, Hospital de Pediatría “Juan P. Garrahan”, Buenos Aires, Argentina; 3 Consejo Nacional de Investigaciones Científicas y Técnicas (CONICET), Buenos Aires, Argentina; University of Bristol, United Kingdom

## Abstract

Polymorphisms in apolipoprotein genes have shown to be predictors of plasma lipid levels in adult cohorts receiving highly active antiretroviral therapy (HAART). Our objective was to confirm the association between the *APOC3* genotype and plasma lipid levels in an HIV-1-infected pediatric cohort exposed to HAART. A total of 130 HIV-1-infected children/adolescents that attended a reference center in Argentina were selected for an 8-year longitudinal study with retrospective data collection. Longitudinal measurements of plasma triglycerides, total cholesterol, HDL-C and LDL-C were analyzed under linear or generalized linear mixed models. The contribution of the *APOC3* genotype at sites −482, −455 and 3238 to plasma lipid levels prediction was tested after adjusting for potential confounders. Four major *APOC3* haplotypes were observed for sites −482/−455/3238, with estimated frequencies of 0.60 (C/T/C), 0.14 (T/C/C), 0.11 (C/C/C), and 0.11 (T/C/G). The *APOC3* genotype showed a significant effect only for the prediction of total cholesterol levels (p<0.0001). However, the magnitude of the differences observed was dependent on the drug combination (p = 0.0007) and the drug exposure duration at the time of the plasma lipid measurement (p = 0.0002). A lower risk of hypercholesterolemia was predicted for double and triple heterozygous individuals, mainly at the first few months after the initiation of Ritonavir-boosted protease inhibitor-based regimens. We report for the first time a significant contribution of the genotype to total cholesterol levels in a pediatric cohort under HAART. The genetic determination of *APOC3* might have an impact on a large portion of HIV-1-infected children at the time of choosing the treatment regimens or on the counter-measures against the adverse effects of drugs.

## Introduction

The introduction of Highly Active Antiretroviral Therapy (HAART) has led to a significant improvement in the survival and life quality of children living with HIV. However, exposure to HAART also leads to some side effects, being body fat redistribution and metabolic abnormalities two recurrent events in treated patients. Antiretroviral (ARV) treatment has been associated with a higher incidence of dyslipidemia, and 20% to 80% of pediatric patients under HAART show high plasma levels of total cholesterol, LDL-C and/or triglycerides [1]–[5]. Since increased plasma lipid levels constitute a risk factor to adulthood cardiovascular disease in healthy children and adolescents [6]–[8], dyslipidemia is not a negligible status and is being increasingly considered at the time of making medical decisions, particularly in patients facing many decades of ARV treatment [9], [10].

Results from genome-wide association studies on the general population suggest that genetic diversity alone accounts for 5–10% of the inter-individual variance observed for lipid traits [11]–[13]. Cross-sectional [14], [15] and longitudinal [16]–[20] studies carried out in HIV-infected patients under HAART associate genetic markers with lipid level shifts. However, these studies have been focused on adult populations and, to our knowledge, there are no published reports on pediatric cohorts.

The gene region of Apolipoprotein C-III (*APOC3*) contains three of the most studied polymorphisms associated with HAART-related dyslipidemia [21]–[23]. The encoded protein is a lipoprotein surface element that inhibits traffic and hydrolysis of lipid particles. The polymorphisms −455T→C and −482C→T are located in the insulin-responsive element (IRE) of the promoter, whereas polymorphism 3238C→G is located in the 3′ untranslated region (3′UTR). White adult patients carrying one or more minority alleles show an additive increase in triglyceridemia in response to HAART, while the opposite is observed in HDL and non-HDL cholesterolemia [14], [17], [23]. To our knowledge, only one report has so far analyzed with enough power the contribution of a gene-treatment interaction to lipid levels in non-White patients under HAART, although the study focused almost exclusively on *APOC3* gene polymorphisms [15]. Collectively, the available evidence supports the fact that *APOC3* is a good candidate to study the significance of a hereditary component in the lipid levels observed in White/Hispanic children under HAART. Thereafter, our aims were to test the association between *APOC3* genetic polymorphisms and plasma lipid levels in an Argentinean pediatric cohort exposed to HAART, and to evaluate the relevance of genotypic data for the prediction of the patient’s lipid profile in a short- and long-term evaluation. An observational and longitudinal study with retrospective data collection was designed to carry out this task, and a repeated measures model was applied for the statistical analysis, taking into account the contribution of potential confounders.

## Materials and Methods

### Ethics Statement

The Ethics Committee and the Review Committee of Clinical Research of the “J.P. Garrahan” Pediatric Hospital, Buenos Aires, Argentina, approved the study. Written informed consent was obtained from the blood donors and the parents or legal guardians of the children.

### Study Design and Settings

A retrospective cohort study was carried out with data collected from January 2001 to December 2008. The sample was composed of White/Hispanic HIV-infected children under HAART, and followed up at the “J.P. Garrahan” Pediatric Hospital. The Hospital is a public referral tertiary care institution located in the City of Buenos Aires, Argentina. Plasma viral load, CD4+ T cell count and standard laboratory lipid determinations were obtained from data routinely recorded on electronic databases, while clinical and demographic data were retrospectively collected from paper-based clinical records.

### Patients

Information on essential fields (e.g. date of birth, sex, estimated date of AIDS onset, use of ARV drugs before HAART initiation) was available for 626 HIV-1-infected children/adolescents who had initiated HAART before December 2008 with protease inhibitors (PI) or non-nucleoside reverse transcriptase inhibitors (NNRTI). All of them had an informed consent from their tutors to perform genetic analysis. All patients with at least two records of plasma lipid levels (>1 month apart) during the study period and at least one stored DNA sample available were included. Thereafter, a final sample of 130 participants was available for genetic characterization. Patients did not receive lipid lowering drugs during the study period or before.

### Definition of Variables

Lipid levels on plasma (mg/dl) were determined repeatedly on blood samples for all patients at irregular time intervals. Patients older than one year old were routinely indicated a 12-hour period of fasting (97.5% of all determinations), although parents/tutor confirmation was not documented. Plasma total cholesterol (TC), triglycerides (TG), high density lipoprotein cholesterol (HDL-C) and low density lipoprotein cholesterol (LDL-C) were quantified by commercially available kits. Dyslipidemia was dichotomously defined for each sample according to the corresponding lipid level observed. Thus, we defined hypercholesterolemia as TC ≥200 mg/dl, hypertriglyceridemia as TG ≥150 mg/dl, low HDL-C as HDL-C ≤40 mg/dl, and high LDL-C as LDL-C ≥130 mg/dl. Longitudinal data on patient age, time since menarche, accumulated time on HAART, drugs indicated on the ongoing treatment regimen, time of exposure to the ongoing treatment regimen, and viral load and CD4+ T cell percentage determinations were collected at the time of each lipid determination. Children’s height and weight were measured at each visit for clinical control, usually on a date close to the blood extraction for plasma lipid analysis. Menarche was considered as a dichotomous variable for further analysis (previous/posterior to menarche). Unknown menarche ages (11% of lipidemia measurements) were extrapolated to the mean observed in HIV-1-infected girls followed at our hospital (12.62 years). AIDS definition was established according to the 1994 criteria of CDC classification for children [24]. AIDS status for participants was defined dichotomously and assessed before the first lipid determination. For the case of eight individuals who progressed to AIDS during the study period, dichotomous AIDS status was defined at the median time point for all patient determinations. Additional patient information that remained unchanged along follow up (cross-sectional variables) included: age at study endpoint, gender, age at HAART initiation, antiretroviral exposure before HAART and APOC3 genotype (Table S1). All patients received HAART, although a high diversity in the nature and number of HAART schemes was observed (Table 1, Table S2). To simplify this variation, only the presence/absence of drugs frequently used in our cohort and with a documented association with lipid levels was analyzed as indicators. Stavudine (D4T), Ritonavir (RTV)-boosted protease inhibitors (PIs), non-nucleoside reverse transcriptase inhibitors (NNRTIs) and Nelfinavir (NFV) were the drugs most commonly included in the regimens indicated at the time of lipid determinations. Among RTV boosted PIs, the most commonly included were Lopinavir (56.4%), Saquinavir (11.4%), and Darunavir (10.2%), while Indinavir, Atazanavir, and Amprenavir were each indicated in less than 6% of all RTV-boosted PIs schemes (Table S2).

**Table 1:** Characteristics of the population studied.

**Cross-sectional description of patients**	
Total number of patients^1^	130
Gender (male/female)	58/72
Age (years) at endpoint^2^ (median and IQR)	14.8 (10.8–16.5)
Years on HAART at endpoint^2^ (median and IQR)	10 (4.8–10.8)
Switches of treatment regimen (median and IQR)	2 (1.75–4)
Lipid determinations per patient (median and IQR)	12 (6–16)
AIDS status (%)	49.2
Initial BMI Z-score (median and IQR)	−0.2 (−1.0–0.8)
**Description at time of lipid determination**	
Total number of lipid determinations	1589
Patients age in years (median and IQR)	12 (8.6–14.4)
Sample collection before menarche age (%)	18.6
%CD4+ T cells^3^ (median and IQR)	27 (19–34)
Viral load (log copies/ml)^4^ (median and IQR)	3.2 (2.2–4.4)
HAART (years) before last regimen (median and IQR)	5.9 (3.5–8.3)
Time (months) on ongoing regimen (median and IQR)	26 (9–51)
Patients receiving D4T (%)	53.9
Patients receiving EFV (%)	28.6
Patients receiving NVP (%)	5.8
Patients receiving PI (1 or more) (%)	68.6
Patients receiving NFV (%)	27.7
Patients receiving PI boosted with RTV (%)	33.2
Patients receiving full dose RTV (%)	7.2
**Longitudinal summary of fasting lipidemia** 5	
Average TG for each patient (median and IQR)	130.5 (93–172)
Patients with any TG ≥150 mg/dl (%)	75.4
Average TC for each patient (median and IQR)	161.5 (145–179)
Patients with any TC ≥200 mg/dl (%)	53.8
Average LDL for each patient (median and IQR)	94.5 (80–121)
Patients with any LDL ≥130 mg/dl (%)	42.2
Average HDL for each patient (median and IQR)	40 (34–49)
Patients with any HDL ≤40 mg/dl (%)	78.3

1 96.2% of patients were born in Buenos Aires, 2.3% in other provinces, and 1.5% in other countries.

2 Study endpoint: December 31^st^, 2008.

3 n = 1484.

4 n = 1574.

5 ≥12 hours fasting status was presumed for 97.5% of blood samples.

### Determination of *APOC3* Genotype and Estimation of Haplotype Frequency

Genomic DNA was extracted from frozen leukocyte pellets by using commercial kits. *APOC3* genetic polymorphisms on the 3′UTR (3238C→G (*rs5128*)) and the IRE region (−455T→C (*rs2854116*) and −482C→T (*rs2854117*)) were respectively resolved by PCR-RFLP with the restriction enzymes SstI, FokI and MspI, as previously described [14], [25]. The haplotype frequencies of the population were estimated by the Expectation Maximization (EM) algorithm from the observed unphased haplotype pairs, as implemented in SNPStats at http://bioinfo.iconcologia.net.

### Statistical Analysis

Longitudinal data on TC, TG, HDL-C, and LDL-C plasma levels were analyzed separately by modeling the independent effects of genetic polymorphisms and additional covariates. The correlation between determinations from the same patient was taken into account by fitting a Linear Mixed (-Effects) Model (LMM), a well-established method for studies with repeated measures. Likewise, longitudinal data analysis on binary outcomes was carried out under a Generalized Linear Mixed Model (GLMM). The predictors and their best functional form were selected in an iterative backward elimination algorithm. Final models were obtained for each lipid, allowing multiple measures per patient, spaced along irregular time intervals, and adjusted for the effect of potential confounders varying along the follow up of each patient. The additional advantages of longitudinal analysis, with focus on the study of ARV-treated patients, have been previously discussed elsewhere [17], [20].

The joint effect of all three *APOC3* SNPs on lipid levels was evaluated with a “global level” likelihood ratio test, with no assumptions on the genetic model. To evaluate any effect of genotype diversity on lipid levels, three different models were postulated as alternatives to the null hypothesis of no genetic contribution (Figure S1): the genotype is associated with the lipid levels regardless of the treatment (alternative 1), the genotype is associated with the lipid levels only under a specific therapeutic scheme (alternative 2), or the genotype shows an association with lipid levels that depends on the accumulated exposure to ARV drugs (alternative 3). Each alternative hypothesis was tested hierarchically, starting with the most general model (alternative 3) against the null hypothesis, and then proceeding with a backward elimination. The interaction between genotype and treatment, included in alternatives 2 and 3, were evaluated only for the interaction with D4T and RTV boosted PIs. The contribution of the terms delineating each postulation was evaluated with hierarchical likelihood ratio tests. Multiple testing corrections were applied for the four different lipids measured, considering that four tests were carried up for each one. Thus, for a family wise error rate below 0.05, Bonferroni corrected tests should reject the null hypothesis with a p-value below 0.003125. For the description of genotype effects, a co-dominant genetic model was fitted to each locus and contrasts were evaluated with a Wald test under the most general model (alternative 3). *APOC3* loci effects were estimated marginally assuming an additive effect. However, the analysis of combined haplotypes showed results with no qualitative differences from those presented here (data not shown). More detail on the models and algorithms applied can be found in the Material and Methods S1 and Algorithm S1.

### Sample Size Considerations

The power to detect PI/genotype interactions was estimated by parametric bootstrapping under the most general LMM. For a statistical test with significance level of 0.003125, the observed frequency of −482T carriers under RTV-boosted PI regimen provided a power greater than 80% to detect differences of 48, 121, 11 and 49 mg/dl on TC, TG, HDL-C and LDL-C plasma levels, respectively. Likewise, for −455C carriers, differences of 44, 109, 11 and 47 mg/dl were respectively detectable. Finally, for 3238G carriers, differences of 48, 132, 12 and 54 mg/dl were detectable.

## Results

### Patients and Characterization of *APOC3* Genotype

A total of 130 children and adolescents were analyzed. A summary of the clinical characteristics of the study participants is depicted in Table 1. Overall, the whole sample showed a wide distribution of age and time on HAART, with a high number of plasma lipid measurements available for each patient.

**Figure 1:**
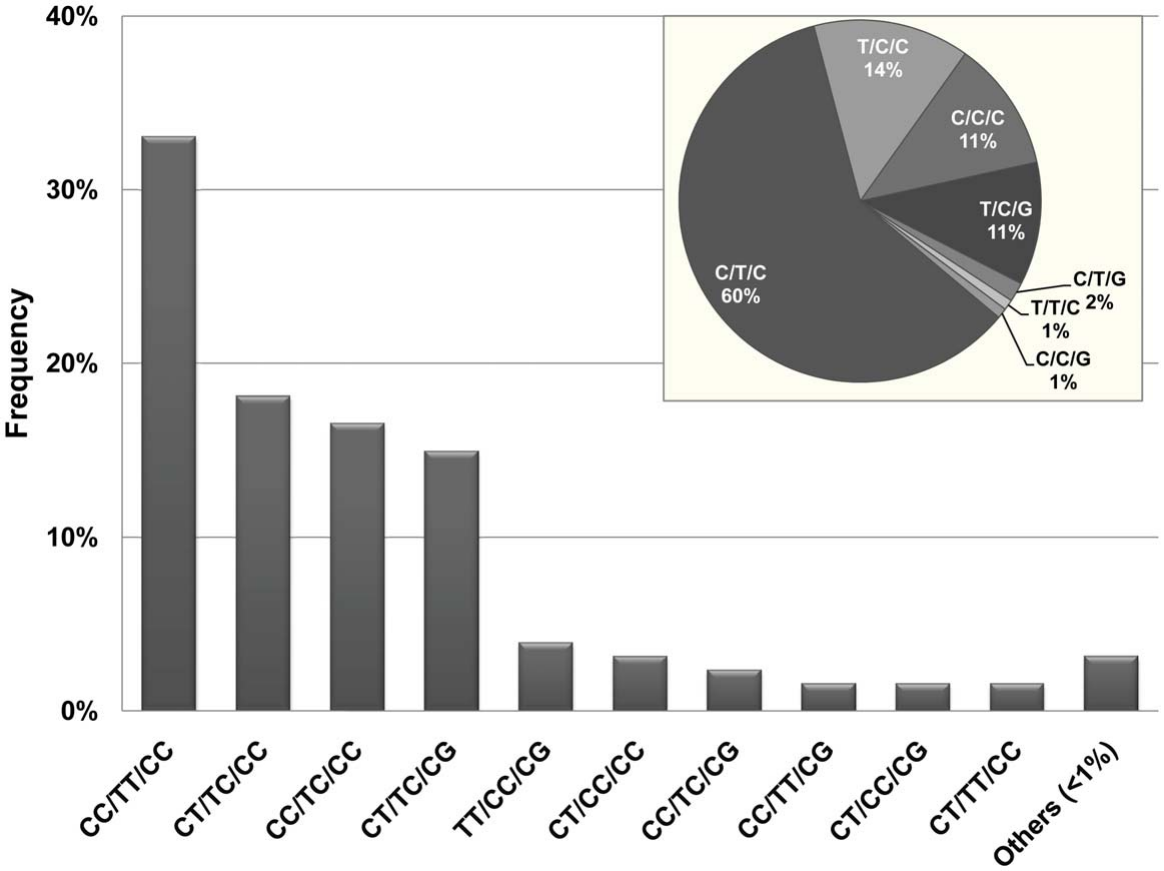
Frequency of haplotype pairs (loci−482, 455 and 3238 on the −*APOC3* gene, respectively) as observed in 127 HIV-1-infected pediatric patients. Inset on top right shows haplotype frequency estimation by an Expectation-Maximization (EM) algorithm.

The genotypic and allelic frequencies of polymorphisms in −482C→T, −455T→C and 3238C→G observed are shown in Table 2. All loci fitted to the Hardy-Weinberg equilibrium. Four major haplotype pairs represented around 83% of the patients, as estimated from 127 fully characterized unphased haplotype pairs (Figure 1). Four major haplotypes represented 96% of the genetic diversity observed, with the haplotype with no minor variants (WT) showing a frequency of 60%. All minor haplotype pairs were observed with frequencies below 5%.

**Table 2:** APOC3 genotypic and allelic frequencies.

	APOC3 *Genotype Frequencies (%)*	
IRE −482 (MspI)	CC	69 (53.5)
	CT	53 (41.1)
	TT	7 (5.4)
	Allele −482T frequency	0.26
IRE −455 (FokI)	TT	46 (35.9)
	TC	68 (53.1)
	CC	14 (10.9)
	Allele −455C frequency	0.38
UTR 3238 (SstI)	CC	96 (74.4)
	CG	31 (24.0)
	GG	2 (1.6)
	Allele 3238G frequency	0.14

### Plasma Lipid Levels in Pediatric Patients on HAART

**Figure 2:**
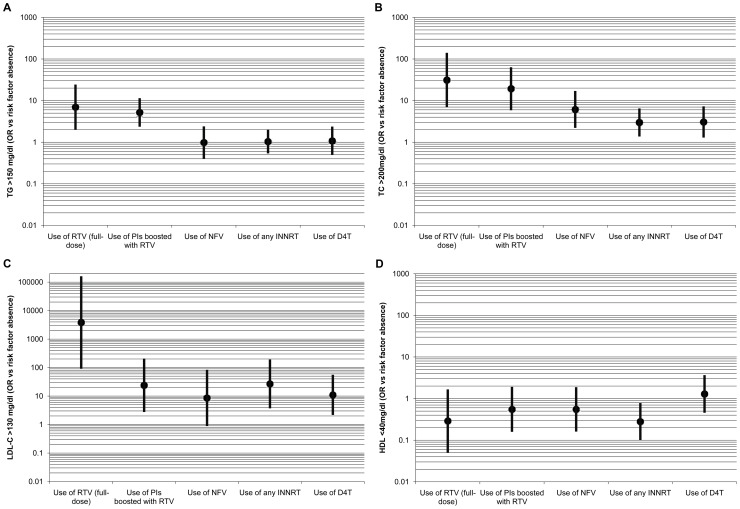
Predicted risk of dyslipidemia according to different treatment options after one year of exposure. Odds ratio (OR) estimates for individuals with no *APOC3* variants (“000” haplotype) were adjusted by the variables listed in Table S1 under Generalized Liner Mixed-effects Model (GLMM), and after backward elimination algorithm. Dots depict punctual estimations for the mean effect of the exposure to each drug while lines depict the 95% confidence intervals.

Data on plasma TC (n = 1589), TG (n = 1578), LDL-C (n = 684) and HDL-C (n = 788) were retrospectively collected from 130 patients (Table 1). Patients with HDL-C/LDL-C data (n = 128) were not significantly fewer than patients with TC and TG, although the number of determinations per patient was markedly reduced to a median of 5 (Inter Quartile Range, IQR: 1–7). One or more events of dyslipidemia, defined as high levels of TC, TG or LDL-C, or low levels of HDL-C, were present in 93.1% of the patients and 86.2% showed at least two events. Patients receiving PI-based regimens including RTV as booster or full dose showed an increased overall frequency of dyslipidemia, mainly for LDL-C and TC determinations. A smaller overall increase was observed in patients on D4T, NNRTI, and NFV (Figure 2).

**Figure 3:**
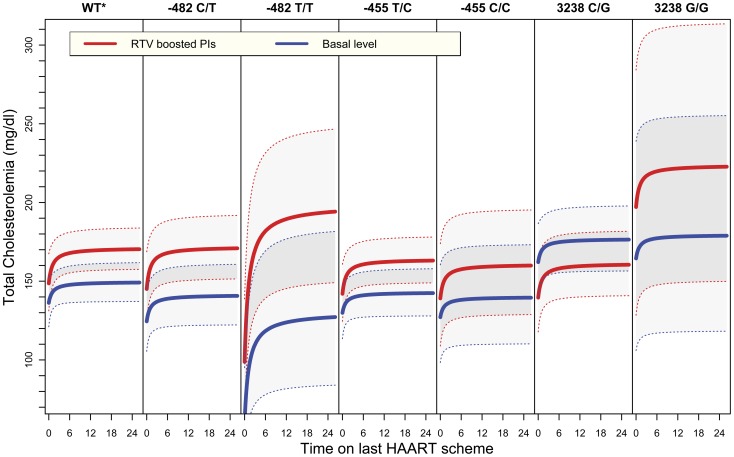
Prediction of mean total cholesterol (TC) plasma levels variations for individuals carrying *APOC3* minor alleles. Linear Mixed-effects Model (LMM) projections for a ARV-experienced male under his first HAART drug regimen. Basal levels for total cholesterolemia were estimated subtracting the effect of adjusted treatment options (RTV, NFV, NNRTIs and D4T) and the effect of minor alleles on other loci. Thick lines depict punctual estimates, whereas dotted lines depict the 95% confidence intervals. *WT =  projections for individuals without any minor alleles.

In order to assess the effect of *APOC3* alleles on lipid levels and risk of dyslipidemia, and to check potential confounders or interactions, the contribution of treatment- and time-dependent factors were collectively evaluated (Table 3). An increase in absolute lipid levels was observed in patients on PI or NNRTI. PIs boosted with RTV and full-dose RTV were the factors with the strongest effect on lipid levels, while a smaller contribution was attributed to NNRTI, NFV and D4T (Figure 2 and Table 3). The final model also depicts a fast increase in the risk to dyslipidemia shortly after treatment initiation (Figure 3), although the time on HAART or the time of exposure to different ARVs only showed a weak correlation to lipid levels (Table 3).

**Table 3:** Prediction of lipids levels under linear mixed models (LMM).

	TG	TC	LDL-C	HDL-C
**Treatment, clinics, demographics**				
After menarche	NA	**0.0030 (–)**	0.1364	NA
HAART exposure before current scheme	0.0061 (+)	0.2185	0.0285 (–)	0.0622
Time on current scheme	0.2472	0.1304	0.0573	0.2397
Age at HAART initiation	NA	NA	NA	0.0148 (+)
Use of RTV (full-dose)	0.0234 (+)	**0.0001 (+)**	**0.0002 (+)**	0.0083 (+)
Use of PIs boosted with RTV	**0.0024 (+)**	**0.0006 (+)**	0.1100	0.3445
Use of D4T	0.4667	0.1224	0.0628	0.9215
Use of NFV	0.5608	0.0037 (+)	0.0932	0.2567
Use of any INNRT	0.0961	0.1160	0.0075 (+)	**0.0011 (+)**
Time on RTV (full-dose) in current scheme	0.5766	0.5055	0.6934	0.8924
Time on PI boosted with RTV in current scheme	0.0285 (+)	0.4141	0.0292 (+)	0.0856
Time on D4T in current scheme	0.3309	0.3742	NA	NA
Time on NFV in current scheme	0.0272 (+)	0.4410	0.5336	0.1643
Time on INNRT in current scheme	0.0070 (+)	0.0757	0.3336	0.4778
**Genotype basal effect^1^**				
UTR 3238 (SsTI) CG vs CC	0.4339	**0.0018 (+)**	NA	0.4168
UTR 3238 (SsTI) GG vs CC	0.3826	0.3652	NA	0.7211
IRE −455 (FokI) CT vs TT	0.9324	0.3931	0.6743	0.9542
IRE −455 (FokI) CC vs TT	0.8022	0.5563	0.1708	0.2396
IRE −482 (MspI) TC vs CC	0.4581	0.3099	0.5593	0.1019
IRE −482 (MspI) TT vs CC	0.1552	0.4468	0.9094	0.9082
**Genotype treatment-associated effect^2^**				
*Effect under RTV boosted PI schemes*				
UTR 3238 (SsTI) CG vs CC	0.0988	**<0.0001 (–)**	NA	0.0968
UTR 3238(SsTI) GG vs CC	0.2560	0.5125	NA	0.4453
IRE −482 (MspI) TC vs CC	NA	0.2046	NA	0.1556
IRE −482 (MspI) TT vs CC	NA	0.0220 (+)	NA	0.8556
*Effect under D4T including schemes*				
UTR 3238 (SsTI) CG vs CC	NA	0.0307 (–)	NA	NA
UTR 3238(SsTI) GG vs CC	NA	0.4041	NA	NA
IRE −455 (FokI) CT vs TT effect	0.4114	0.2845	0.9933	0.0332 (–)
IRE −455 (FokI) CC vs TT effect	0.0606	0.0488 (+)	0.0315 (+)	0.8261
IRE −482 (MspI) TC vs CC effect	NA	0.2354	NA	0.0573
IRE −482 (MspI) TT vs CC effect	NA	0.0459 (–)	NA	0.8362
**Genotype effect associated with exposure time^3^**				
*Interaction with accumulated HAART time*				
IRE −455 (FokI) CT vs TT	NA	0.1992	0.7722	NA
IRE −455 (FokI) CC vs TT	NA	0.5146	0.3264	NA
IRE −482 (MspI) TC vs CC	0.0734	NA	NA	0.0702
IRE −482 (MspI) TT vs CC	0.4530	NA	NA	0.9457
*Interaction with time on current scheme*				
IRE −455 (FokI) TC vs CC	NA	NA	NA	0.0874
IRE −455 (FokI) TT vs CC	NA	NA	NA	0.9317
IRE −482 (MspI) TC vs CC	0.0238 (–)	0.6402	0.9263	0.7546
IRE −482 (MspI) TT vs CC	0.8174	**<0.0001 (+)**	0.0364 (–)	0.4226

The contribution of each factor was evaluated with Wald test on 127 individuals with full haplotype characterization; p-values are depicted. Significant p-values (p<0.003125, after Bonferroni correction) are indicated in bold numbers. Correlation sign is depicted between parentheses for p-values below 0.05. NA: variable excluded by stepwise backward elimination. ^1^Alternative 1 (Figure S1). ^2^Alternative 2 (Figure S1). ^3^Alternative 3 (Figure S1).

Since cholesterol levels of patients with AIDS status were not significantly different from those of patients who presented a more favorable disease course (p>0.02), the final model did not adjust to AIDS. The contribution of longitudinal measures of viral load, CD4+ T cell counts and Body Mass Index (BMI) to the significances observed was evaluated separately. Their addition made only a negligible shift in the contribution of qualitative treatment indicator variables (i.e. use of RTV, D4T, NFV or NNRTI) and genotype (Table S3 and Table S5). Further diagnoses of model assumptions were described in the Results S1, Figure S2 and Figure S3.

### *APOC3* Diversity is Associated with Absolute Total Cholesterol Plasma Level Variation

We first evaluated whether knowledge on the *APOC3* genotype -IRE −482, IRE −455 loci and 3′UTR 3238- can predict lipid plasma concentrations in children/adolescents under HAART. With this aim, *APOC3* association with lipid levels was evaluated for the combined effect of *APOC3* loci on TC, TG, HDL-C and LDL-C plasma levels (Table 4, Alternative 3 vs Null). The genotype showed a significant effect for the total cholesterol model. There was no evidence of mean basal (treatment independent) lipid level differences among *APOC3* genotypes (Alternative 1). In contrast, the interaction between genotype and treatment showed a significant contribution to the model, suggesting that *APOC3* genetic diversity is related to the impact of HAART on the patient, particularly for PIs schemes boosted with RTV and D4T (Alternative 2). Moreover, a time-dependent association of *APOC3* was observed, suggesting a systematic variation with patient age or time of exposure to ARV drugs (Alternative 3). On the other hand, there was no statistical evidence of the influence of *APOC3* diversity on any alternative model for TG, LDL-C or HDL-C (Table 4).

**Table 4:** Hierarchical test for differences in absolute plasma levels (LMM)^1^.

	TG	TC	LDL-C	HDL-C
**Effect of ** ***APOC3*** ** genotype** 2	0.1176	<.0001	0.2681	0.2319
**Genotype effect associated with exposure time** 3	0.1495	0.0002	0.2563	0.2791
**Genotype effect associated with treatment** 4	0.2298	0.0007	0.2758	0.3144
**Genotype basal effect** 5	0.2368	0.1672	0.3824	0.2766

1 Bonferroni corrected significance level was α* = 0.003125.

2 Test for the contribution of *APOC3* genotypes taking into account interactions with specific treatment scheme (inclusion of PIs boosted with RTV and/or D4T) and time of exposure (alternative 3 vs. null, Figure S1).

3 Test for the contribution of the interaction between *APOC3* genotypes and time of exposure (alternative 3 vs. alternative 2).

4 Test for the contribution of the interaction between *APOC3* genotypes and treatment scheme (alternative 2 vs. alternative 1).

5 Test for the contribution of *APOC3* genotypes without interaction (alternative 1 vs. null).

### The Association between the *APOC3* Genotype and Total Cholesterol Levels is Explained by a Differential Response to ARV Exposure Rather than by Fixed Genetic Differences Alone

The association found between *APOC3* and TC levels was explored in more detail to disclose the genotype contrasts responsible for the significance observed. The projected TC level variations in response to PI regimens boosted with RTV among *APOC3* genotypes are depicted in Figure 3. An increase in plasma cholesterol levels was observed for most genotypes shortly after the initiation of PI regimens boosted with RTV. While patients with genotypes −482 CT, −482 TT and −455 TC kept the same trend observed for wild type individuals (carrying no minor alleles), patients with genotypes −455 CC, 3238 CG and 3238 GG showed a milder variation after regimen initiation. The strongest allele effect was observed for 3238 CG heterozygous individuals, where TC levels projections before and after initiation of PI schemes boosted with RTV were similar (p<0.0001, Table 3). Additionally, −482 genotype showed a trend indicating an association with cholesterolemia under RTV-boosted PI, but with no statistical significance after Bonferroni correction (p = 0.0220). A weak statistical signal was also confirmed for patients under D4T on the three loci studied (Table 3). Likewise, *APOC3* polymorphisms were also associated with the effect of HAART on children after different times of exposure. In agreement to the mean TC levels observed under qualitative treatment variations, IRE −482 TT genotype showed a marked increase in cholesterolemia over time, in contrast to IRE −482 CC homozygous genotype.

Despite the lack of confirmatory evidence of a contribution of the *APOC3* genotype to plasma LDL-C or HDL-C level variation, the fitted models showed some trends with no statistical significance (Table 3).

### Impact of *APOC3* Haplotype Variation on the Prediction of Hypercholesterolemia

**Figure 4:**
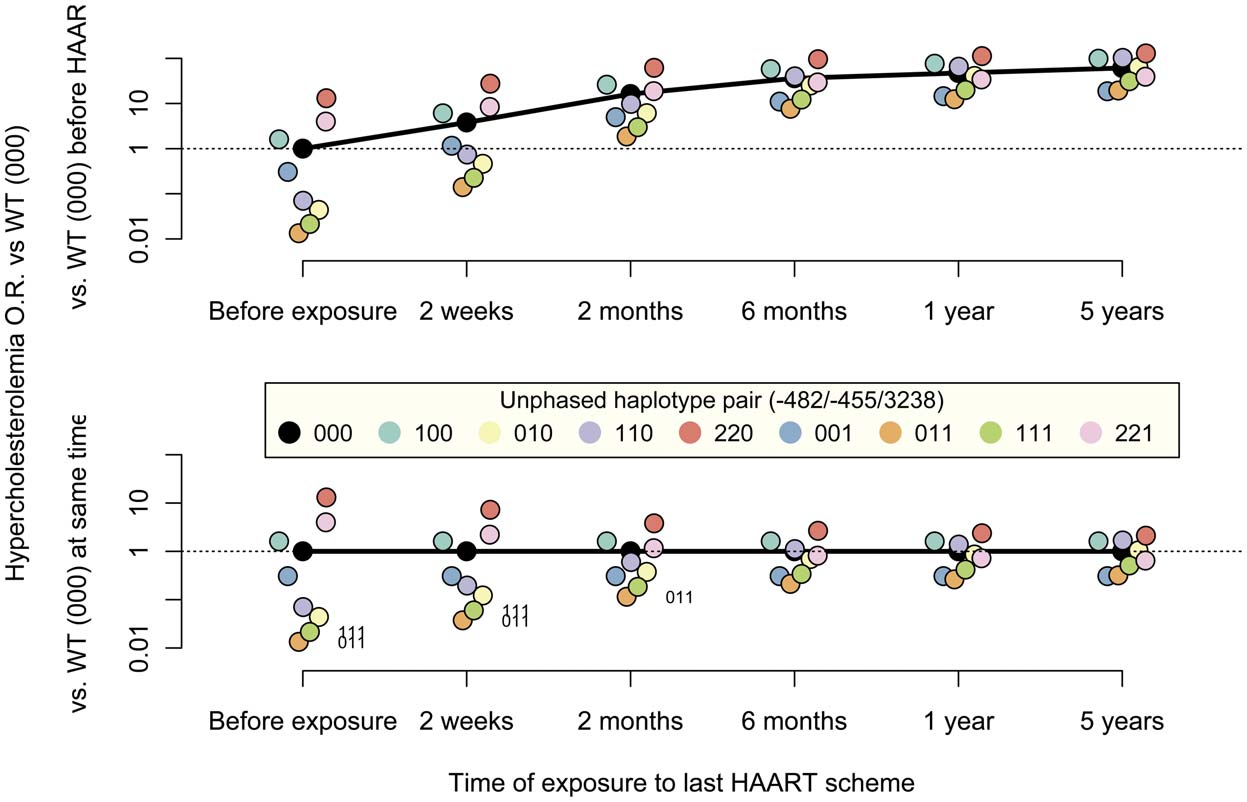
Generalized Linear Mixed-effects Model (GLMM) projections of hypercholesterolemia risk for patients carrying the indicated haplotype pairs. GLMM projections for a ARV-experienced male (treatment regimen starting after 62 months on HAART, the observed mean time on HAART at new regimen initiation) under a RTV-boosted PI regimen without D4T. Upper and lower panels depict contrasts to WT before exposure or at the same exposure time. Haplotype notation indicates the gene dose at each locus. Dots represent punctual contrast projections. Dots with labels indicate haplotype pairs statistically significantly different from WT at the same time (only lower panel). Odds ratios for common haplotype pairs “020”, “120” and “121” could not be estimated due to power issues. The contribution of all the factors included in GLMM were included in Table S4.

In order to evaluate whether the observed association of *APOC3* genotype with TC plasma levels had clinical relevance by significantly changing the susceptibility to HAART-associated hypercholesterolemia, we estimated the risk for each haplotype pair (Figure 4). As anticipated, the risk to abnormal high TC levels increased with the time of exposure to HAART. The contrasts among haplotypes within the same time of exposure showed a significant difference from the wild type −482CC/−455TT/3238CC (“000”) for −482CC/−455TC/3238CG (“011”) and −482TC/−455TC/3238CG (“111”). Nevertheless, this difference was statistically significant only for exposure times shorter than 6 months. Thus, patients with longer times of exposure to HAART showed an increased risk of hypercholesterolemia and differences attributable to haplotypes became less evident. Conversely, model projections for patients with little or no exposure to HAART showed a lower risk of increased TC levels, but with more evident differences among haplotypes. In summary, there was evidence for a differential risk of HAART-associated dyslipidemia conditioned by the *APOC3* haplotype pair. However, increasing time of exposure to HAART diminished the differences among haplotypes, as the chance to hypercholesterolemia reached a common ceiling.

## Discussion

### Key Findings

We found an association between *APOC3* gene polymorphisms and TC levels in HIV-1-infected children/adolescents under HAART. We also predicted a lower risk of hypercholesterolemia for double and triple heterozygous individuals in loci −482, −455 and 3238. The differences observed seemed dependent on the drug combination, being more evident in patients under PI-based regimens boosted with RTV. However, model projections depicted an effect for *APOC3* haplotype that was more significant shortly after HAART initiation than later on. This is the first report of a significant interaction between *APOC3* gene effect on lipid levels and drug exposure time. No association was found between *APOC3* gene polymorphisms and TG, LDL-C or HDL-C plasma levels.

This work is an extension and confirmatory study of previous reports stating an association between genetic polymorphisms and lipid levels in adult patients under HAART [14], [15], [17], [23]. To our knowledge, this is the first study in a pediatric cohort, as well as the only one for Argentina and Latin America.

### Global Effect of the *APOC3* Genotype on the Prediction of Lipid Levels

*APOC3* genotypes showed different total cholesterol levels, and these differences were conditional to the drugs indicated. The influence of *APOC3* gene polymorphisms is in agreement with a decrease in HDL or non-HDL cholesterolemia for the minor allele observed in previous studies on ARV-treated adult cohorts [14], [17], [23]. However, in the pediatric cohort of this study, a decrease in plasma levels was evident for total cholesterol, but not for any cholesterol component, Since ethnicity has been postulated as a confounder for the *APOC3* effect on lipid levels observed [15], the comparison of our results with those of other studies might be hampered by dissimilar genetic compositions. Argentina’s population is composed mainly of an admixture of Amerindian and European ancestry [26], and genetic associations may not be directly extrapolated from other studies on individuals with a predominant White component. Moreover, an effect from undetermined genetic polymorphisms might confound the association observed between lipidemia and *APOC3* gene polymorphisms. In particular, Tarr *et al*. [17] found a significant interaction between APOC3 and APOE on the prediction of TG levels. Similarly, interactions between *APOC3* and *APOA5* have been reported [27]. However, a marginal effect of *APOC3* has even been observed in studies that omitted *APOE* and/or *APOA5* determinations, or that did not show significant epistasis [14]–[16]. Discrepancies from previous studies could also be explained by characteristics inherent to the medical care of pediatric patients. As it is well known that the impact of PI on lipid metabolism varies among drugs, a variable strength for the interaction between *APOC3* genetic polymorphisms and lipid levels could be expected from a different composition of ARV drugs. Particularly, RTV sparing treatment with NFV represented 40% of the HAART schemes in this study, while adult patients in previous studies occasionally received this ARV [14].

A novel observation was that *APOC3* gene effect is also conditioned by the time of exposure to HAART. Many features exclusive of our cohort, such as children metabolic development or environmental variations, might be the cause of this observation. Since this was a retrospective and observational study, this interaction was prone to be confounded by lurking historical trends, data collection bias or patient adherence differences. However, whether the observed interactions were inherent to the pediatric population or to limitations from a retrospective study design, the influence of time on *APOC3* gene participation in the prediction of TC levels was an exploratory finding that may explain differences in previous discordant results and merits to be taken into account for the design of future studies.

A noticeable discrepancy from previously published cohorts was the absence of an association between *APOC3* genotype and TG plasma levels [14], [16], [17], [23]. A low power to detect differences in TG plasma levels was estimated *a posteriori* from the genotype frequencies observed and fitted LMM, enough to detect only a difference of 109 mg/dl or greater for minor allele carriers under PI. This range is close to the effect reported by Fauvel *et al.* for *APOC3* polymorphisms in White treated adults [14], although two to five times higher than that reported in other White or Hispanic populations [15]–[17]. Although the number of children/adolescents included in this study was limited, the low power obtained could be explained by a high dispersion rather than by the small sample size alone, since the number of patients included was larger than that reported in previous studies finding a significant association between the same *APOC3* polymorphisms and TG levels [14], [15]. The increased variability of TG levels observed may be due to a high sensitivity to lifestyle, ARV treatment, clinical status, or fasting status, although the precise cause could not be pinpointed in the pediatric population studied.

### *APOC3* Genotype and Total Cholesterol Plasma Levels Dynamics

A co-dominant genetic model was fitted for *APOC3* sites −482, −455 and 3238, and in all cases we found lower hypercholesterolemia risk for heterozygous individuals than for patients without the minor allele under a PI-based regimen boosted with RTV. Model-based projections for hypercholesterolemia events pointed out two haplotypes with a significantly reduced risk. Patients under RTV-boosted treatment regimens who carried two or three minor alleles, specifically −482CC/−455TC/3238CG and −482CT/−455TC/3238CG, showed a lesser chance to an event than patients without any minor allele −482CC/−455TT/3238CC. The trend for these *APOC3* loci was confirmatory of the genetic model previously proposed for adults under PI treatment [17], [23]. The observed effect of −482T or −455C on TC levels could be related to an *APOC3* transcription deregulation, since both polymorphisms are located in a putative insulin response element and their presence may abolish insulin modulation of *APOC3* [28]. On the other hand, there are no functional studies that give a molecular basis for the effect of 3238G. Alternatively, observed associations cannot be discarded as indirect evidence of neighboring genetic markers.

In this pediatric cohort, the evidence for the beneficial effects of *APOC3* minor variants on the risk of hypercholesterolemia was limited to an early period after initiation of PI treatment boosted with RTV, and was not verifiable six months later. This trend occurred jointly with a rapid risk increase, common to all haplotypes, and a sudden increase in the predicted cholesterolemia. Although the *APOC3* genotype may still contribute to cholesterol levels after a long-term exposure, dyslipidemia attributed to HAART would be strong enough to conceal subtle genotype differences. Additionally, a level plateau, due for instance to a host homeostatic response, could result in a reduced margin for further variation. This last scenario would be in agreement for the effect observed for 3238 CG heterozygous patients, who showed the highest background TC levels and the lowest increase after boosted PI treatment. Nevertheless, a cautious interpretation is advised before an external validation is performed, since the estimated dynamics may reflect an indirect effect of lurking variables coupled to time.

A longitudinal model was applied to analyze repeated measures on each patient, while taking into account multiple intervining factors. This represents a powerful method to quantitate the contribution of genetic factors in the context of complex traits [17]. A discussion on the election of the statistical approach can be found in the Discussion S1.

### Study Extrapolations and Clinical Implications

The kinetics and dynamics of ARV drugs show a wide variation among HIV-1-infected patients but, in contrast, HIV-1 therapy is bounded to a narrow edge to accomplish efficiency and safety. The raising possibility of a patient-tailored treatment of HIV-1 infection promise more predictable outcomes. In particular, genetics seem to play a decisive role in fitting therapy to HIV-1-infected children, due to the difficulties of conducting pharmacokinetic/pharmacodynamic studies in this population. Our results predicted a difference in TC levels among common haplotypes in pediatric patients under HAART, mainly in regimens containing RTV. Thus, *APOC3* genetic determination might have an impact on a large portion of HIV-1-infected children, either on the design of antiretroviral therapy or on the set up of counter-measures against the secondary effects of HAART.

Our findings support the association between APOC3 genotype and TC levels observed in HIV-1-infected adults under HAART and are also in agreement with the proposed genetic model for these loci. Future perspectives of this work include the determination of additional genetic polymorphisms associated with lipid metabolism, and the measurements of apolipoprotein plasma levels, to further specify the dynamics of HAART-associated dyslipidemia in children/adolescents infected with HIV-1.

## Supporting Information

Figure S1 **Construction of alternative hypothesis for hierarchical testing.** Figure depicts the variables effect estimated on each model (see Table S1).
(TIF)

Figure S2 **Control for the distribution of likelihood ratio test statistics under the null hypothesis.** Alternative 3 vs Null hypothesis (Figure S1) test for the model fitted to TC levels is depicted here as an example. **A)** Nominal p-values were simulated for chi squared distribution with 19, 20 and 21 degrees of freedom (nominal degrees of freedom for this test was 20), and an equal weights mix of this 3 distributions. Nominal and empirical p-values were generated with the R package *nlme*. Briefly, new data was simulated 1000 times under parameter values as estimated in the null model from real data, with no intra cluster correlation. Then, null and alternative models were fitted to generated data, and likelihood ratio test was carried out for each simulation. Nominal p-values were obtained assuming a chi square distribution for the double of the difference between the two fitted likelihoods. Assumed degrees of freedom were alternated to search for the best fit to p-values empirical distribution –this last defined as the quantile for p-values of all the simulated tests-. The degrees of freedom that best fit empirical p-values may not be nominal, as in this example case. A perfect fit would show a straight identity line. **B)** Likelihood ratio tests for TC levels model, assuming test statistic distributions with alternative degrees of freedom. Significance obtained with the empirical “fittest” degrees of freedom –when fittest were not the nominal degrees of freedom- had not a difference bigger than 1 order and did not show a qualitative change for results in any test carried, as in this example.
(TIF)

Figure S3 **Prediction of mean TC plasma levels variations for individuals carrying APOC3 minor alleles.** Most parsimonious functional form for each continuous scale predictors was chosen following second degree fractional polynomials algorithm. LMM projections for a treatment experienced male under his first HAART drug scheme. Basal levels for total cholesterolemia were estimated subtracting the effect of adjusted treatment options (RTV, NFV, NNRTIs and D4T). Thick line depicts punctual estimation and dotted lines, 95% confidence intervals.
(TIF)

Table S1 **Measured variables and funtional form for analysis.**
(PDF)

Table S2 **Detailed drugs combinations at the time of lipids determinations.**
(PDF)

Table S3 **Prediction of lipids levels from the Linear Mixed-effects Model (LMM) including viral load and T CD4+ cell count.**
(PDF)

Table S4 **Prediction of lipids levels from the Generalized Linear Mixed-effects Model (GLMM).**
(PDF)

Table S5 **Hierarchical test for absolute plasma levels difference under alternative LMM models for total cholesterol levels.**
(PDF)

Algorithm S1 **Multiple fractional polynomials algorithm applied for model building.**
(PDF)

Discussion S1 (PDF)

Material and Methods S1 (PDF)

Results S1 (PDF)
