# Supplementary figures and images for: Hypercholesterolemia Is Associated with the Apolipoprotein C-III (APOC3) Genotype in Children Receiving HAART: An Eight-Year Retrospective Study
Source: PLoS One. 2012 Jul 25;7(7):e39678. doi: 10.1371/journal.pone.0039678 (PMC3405089; doi:10.1371/journal.pone.0039678)

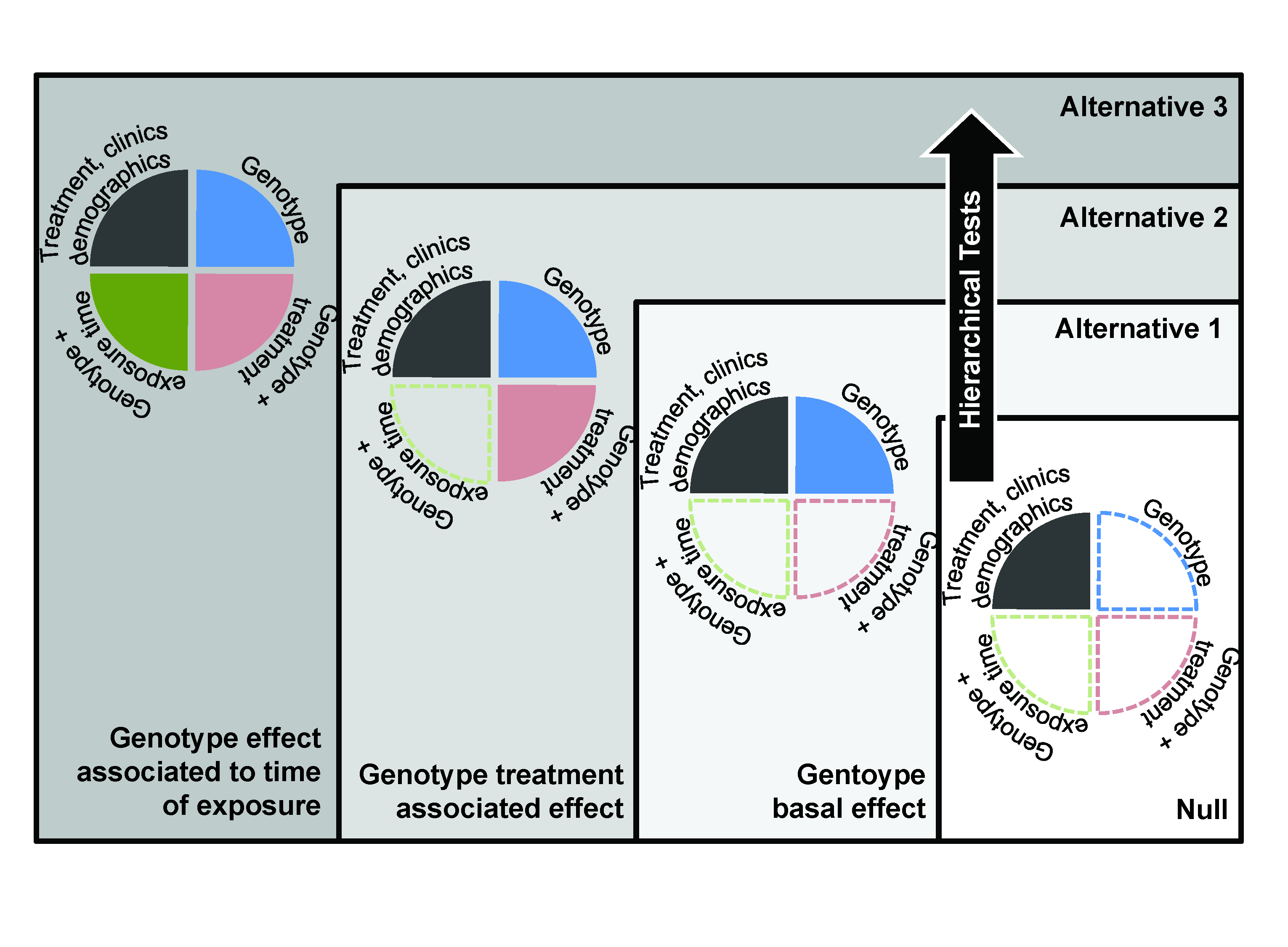

Supplement: Figure S1 — Construction of alternative hypothesis for hierarchical testing. Figure depicts the variables effect estimated on each model (see Table S1). (TIF) [file pone.0039678.s001.tif]

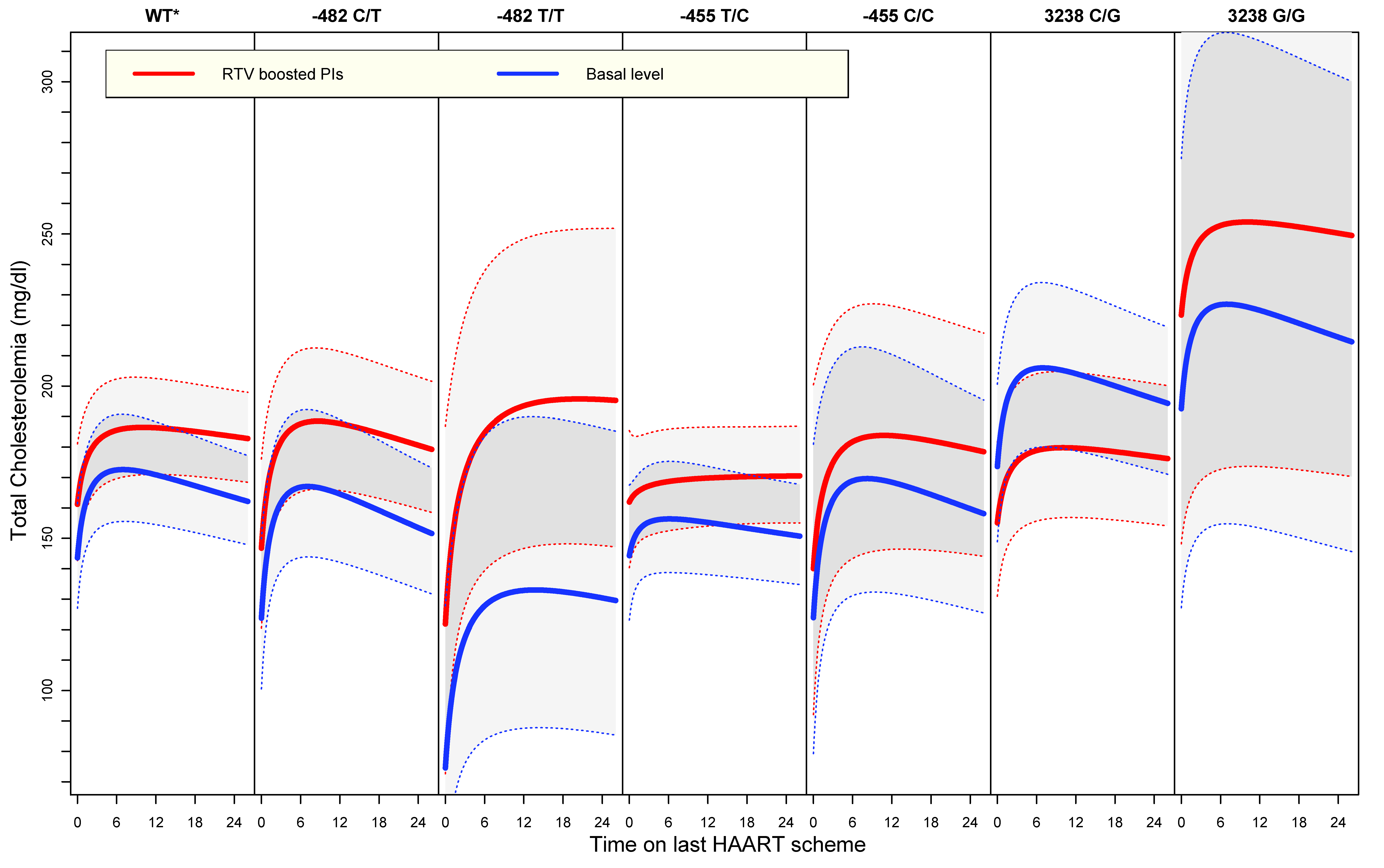

Supplement: Figure S3 — Prediction of mean TC plasma levels variations for individuals carrying APOC3 minor alleles. Most parsimonious functional form for each continuous scale predictors was chosen following second degree fractional polynomials algorithm. LMM projections for a treatment experienced male under his first HAART drug scheme. Basal levels for total cholesterolemia were estimated subtracting the effect of adjusted treatment options (RTV, NFV, NNRTIs and D4T). Thick line depicts punctual estimation and dotted lines, 95% confidence intervals. (TIF) [file pone.0039678.s003.tif]
