# Supplementary material for: Hypercholesterolemia Is Associated with the Apolipoprotein C-III (APOC3) Genotype in Children Receiving HAART: An Eight-Year Retrospective Study
Source: PLoS One. 2012 Jul 25;7(7):e39678. doi: 10.1371/journal.pone.0039678 (PMC3405089; doi:10.1371/journal.pone.0039678)
Supplement: Table S1 — Measured variables and funtional form for analysis. (PDF) [file pone.0039678.s004.pdf]

Supplementary Table S1. Measured variables and funtional form for analysis.

Measured Variable	Starting model for fractional polynomial algorithm	Variable type	Hierarchical model term[1]
Cross-sectional variables (patient invariant)			
Date of birth	Age at HAART initiation (months)	Continuous	Null
Sex	Sex	Binary	Null
Date of AIDS onset	AIDS before first lipid determination	Binary	Null
Use of ARV drugs before HAART start	Naive status	Binary	Null
APOC3 Haplotype	IRE -482 CC/TC/TT	Categorical[2]	Alternative 1
	IRE -455 TT/TC/CC	Categorical[2]	Alternative 1
	3'UTR 3238 CC/CG/GG	Categorical[2]	Alternative 1
Longitudinal variables (time variant)			
Time to first HAART scheme initiation	Time on HAART before current scheme (months)	Continuous	Null
Time on current scheme	Time on current scheme	Continuous	Null
Current scheme drugs	Use of RTV (full-dose)	Binary	Null
	Use of RTV-boosted PI treatment	Binary	Null
	Use of NFV	Binary	Null
	Use of any NNRTI	Binary	Null
	Use of D4T	Binary	Null
Plasma lipids levels	Triglycerides (mg/dl)	Continuous	Response
	Total Cholesterol (mg/dl)	Continuous	Response
	LDL-C (mg/dl)	Continuous	Response
	HDL-C (mg/dl)	Continuous	Response
Viral load close to lipids determination	Viral load	Continuous	Null
CD4+ T cell count close to lipids determination	CD4+ T cell count (%)	Continuous	Null
Weight/height close to lipids determination	BMI Z-score	-	-
Date of menarche	Before/after menarche	Binary	Null
Interactions			
	Time on Current scheme drugs	Continuous	Null
	APOC3 genotype and current scheme drugs (only for D4T and RTV)	Categorical	Alternative 2
	Time on current scheme and APOC3 genotype	Continuous	Alternative 3
	Time on HAART before current scheme and APOC3 genotype	Continuous	Alternative 3

List of all variables as they were measured, and as included in LMM/GLMM.

[1] least general model where variable is included (Supplementary Figure S1)

[2] two contrasts versus wildtype (most frequent allele)
