# Supplementary material for: Hypercholesterolemia Is Associated with the Apolipoprotein C-III (APOC3) Genotype in Children Receiving HAART: An Eight-Year Retrospective Study
Source: PLoS One. 2012 Jul 25;7(7):e39678. doi: 10.1371/journal.pone.0039678 (PMC3405089; doi:10.1371/journal.pone.0039678)
Supplement: Table S2 — Detailed drugs combinations at the time of lipids determinations. (PDF) [file pone.0039678.s005.pdf]

Supplementary Table S2. Detailed drugs combinations at the time of lipids determinations.

PI scheme	-NNRTI	+NNRTI[1]	Total
**RTV-Boosted PI[2]**	**449 (28.3)**	**78 (4.9)**	**527 (33.2)**
-D4T	280 (17.6)	40 (2.5)	320 (20.1)
+D4T	169 (10.6)	38 (2.4)	207 (13.0)
**NFV**	**399 (25.1)**	**41 (2.6)**	**440 (27.7)**
-D4T	141 (8.9)	5 (0.3)	146 (9.2)
+D4T	258 (16.2)	36 (2.3)	294 (18.5)
**PI (not NFV)**	**120 (7.6)**	**3 (0.2)**	**123 (7.7)**
-D4T	48 (3.0)	1 (0.1)	49 (3.1)
+D4T	72 (4.5)	2 (0.1)	74 (4.7)
**no PI**	**29 (1.8)**	**425 (26.7)**	**454 (28.6)**
-D4T	20 (1.3)	152 (9.6)	172 (10.8)
**Total**	**997 (62.7)**	**547 (34.4)**	**1544 (97.2)**
**-D4T**	489 (30.8)	198 (12.5)	687 (43.2)
**+D4T**	508 (32.0)	349 (22.0)	857 (53.9)
**no treatment**			**45 (2.8)**

Number of observed drug combinations (%) during patients' follow up.

[1] 455 (83.2%) and 92 (16.8%) drug combinations included Efavirenz and Nevirapine, respectively, from 547 including NNRTI

[2] 297 (56.4%) included Lopinavir, 81 (15.4%) Saquinavir, 60 (11.4%) Darunavir, 54 (10.2%) Indinavir, 31 (5.9%) Atazanavir, and 30 (5.7%) Amprenavir, from 527 drug combinations including PIs boosted with RTV
