# Supplementary material for: Hypercholesterolemia Is Associated with the Apolipoprotein C-III (APOC3) Genotype in Children Receiving HAART: An Eight-Year Retrospective Study
Source: PLoS One. 2012 Jul 25;7(7):e39678. doi: 10.1371/journal.pone.0039678 (PMC3405089; doi:10.1371/journal.pone.0039678)
Supplement: Table S3 — Prediction of lipids levels from the Linear Mixed-effects Model (LMM) including viral load and T CD4+ cell count. (PDF) [file pone.0039678.s006.pdf]

Supplementary Table S3. Prediction of lipids levels from the Linear Mixed-effects Model (LMM) i[illegible] load and T CD4+ cell count.

Variable	Triglycerides	Total Chol	LDL-C[1]
**Treatment, clinics, demographics (Null model)**			
T CD4+ cells (%)	**0,0004** (-)	0,1550	0,0540
Viral load (copies/ml)	0,0207 (-)	**<0.0001** (-)	**<0.0001** (-)
After menarche	NA	**0,0014** (-)	0,0491 (-)
HAART exposure before current scheme	0,0178 (+)	0,3230	0,0562
Time on current scheme	0,1788	0,2920	0,0202 (-)
Age at HAART initiation	NA	NA	NA
Naive status when starting HAART	NA	NA	NA
Use of RTV (full-dose)	0,0597	**<0.0001** (+)	**0,0005** (+)
Use of PIs boosted with RTV	0,0053 (+)	**0,0001** (+)	0,2487
Use of D4T	0,4291	0,0368 (+)	0,0593
Use of NFV	0,5722	**0,0005** (+)	0,0548
Use of any INNRT	0,1066	0,0768	0,0391 (+)
Time on RTV (full-dose) in current scheme	0,4582	0,2120	0,4452
Time on PI boosted with RTV in current scheme	0,0481 (+)	0,2920	0,0200 (+)
Time on D4T in current scheme	0,2673	0,4690	NA
Time on NFV in current scheme	0,0318 (+)	0,1930	0,4203
Time on INNRT in current scheme	0,0119 (+)	0,1720	0,6840
**Genotype basal effect**			
UTR 3238 (SsTI) CG vs CC	0,3552	**0,0010** (+)	NA
UTR 3238 (SsTI) GG vs CC	0,4326	0,3710	NA
IRE -455 (FokI) CT vs TT	0,5779	0,6690	0,9816
IRE -455 (FokI) CC vs TT	0,3008	0,4440	0,1693
IRE -482 (MspI) TC vs CC	0,7988	0,3130	0,9461
IRE -482 (MspI) TT vs CC	0,5927	0,5800	0,5546
**Genotype treatment-associated effect**			
Effect under RTV boosted PI shemes			
UTR 3238 (SsTI) CG vs CC	0,1364	**<0.0001** (-)	NA
UTR 3238(SsTI) GG vs CC	0,3115	0,6750	NA
IRE -482 (MspI) TC vs CC	NA	0,2290	NA
IRE -482 (MspI) TT vs CC	NA	0,0519	NA
Effect under D4T including shemes			
UTR 3238 (SsTI) CG vs CC	NA	0,0231 (-)	NA
UTR 3238(SsTI) GG vs CC	NA	0,4820	NA
IRE -455 (FokI) CT vs TT effect	0,3546	0,6630	0,5179
IRE -455 (FokI) CC vs TT effect	0,1126	0,0566	0,0206 (+)
IRE -482 (MspI) TC vs CC effect	NA	0,4720	NA
IRE -482 (MspI) TT vs CC effect	NA	0,0591	NA
**Genotype effect associated to time of exposure**			
Interaction with accumulated HAART time			
IRE -455 (FokI) CT vs TT	NA	0,4180	0,7955
IRE -455 (FokI) CC vs TT	NA	0,5210	0,6491
IRE -482 (MspI) TC vs CC	0,0886	NA	NA
IRE -482 (MspI) TT vs CC	0,5225	NA	NA
Interaction with time on current scheme			
IRE -455 (FokI) TC vs CC	NA	NA	NA
IRE -455 (FokI) TT vs CC	NA	NA	NA

IRE -482 (MspI) TC vs CC	0,0239 (-)	0,6670	0,8108
IRE -482 (MspI) TT vs CC	0,8296	**<0.0001** (+)	0,0227 (-)

The contribution of each factor was evaluated with Wald test on 127 individuals with full haplotype characterization; p-values are depicted. Significant p-values (p<0.003125, after Bonferroni correction) indicated in bold numbers.Correlation sign is depicted between parentheses for p-values below 0.05. excluded by stepwise backward elimination.

[1] 3238 homozygous individuals(n=2) excluded before model construction

ncluding viral

HDL-C[1]	
0,0098	(-)
**<0.0001**	(-)
NA	
0,0482	(-)
0,4629	
0,1301	
NA	
**0,0003**	(+)
0,0318	(+)
0,7538	
0,1852	
0,0042	(+)
0,5930	
0,3745	
NA	
0,1755	
0,2603	
0,3549	
NA	
0,7164	
0,1220	
0,2598	
0,9087	
0,1908	
NA	
0,0763	
0,5709	
NA	
NA	
0,0067	(-)
0,8165	
0,0207	(+)
0,8937	
NA	
NA	
0,0550	
0,9081	
0,2798	
0,7255	

0,7398
0,6230

) are
NA: variable
