# Supplementary material for: Hypercholesterolemia Is Associated with the Apolipoprotein C-III (APOC3) Genotype in Children Receiving HAART: An Eight-Year Retrospective Study
Source: PLoS One. 2012 Jul 25;7(7):e39678. doi: 10.1371/journal.pone.0039678 (PMC3405089; doi:10.1371/journal.pone.0039678)
Supplement: Table S4 — Prediction of lipids levels from the Generalized Linear Mixed-effects Model (GLMM). (PDF) [file pone.0039678.s007.pdf]

Supplementary Table S4. Prediction of lipids levels from the Generalized Linear Mixed-effects Model (GLMM).

Hypercholesterolemia model	p value (correlation sign)
**Treatment, clinics, demographics (Null model)**	
After menarche	0,0079 (-)
HAART exposure before current scheme	0,0287
Time on current scheme	0,0287 (-)
Use of RTV (full-dose)	<0.0001 (+)
Use of RTV-boosted PI treatment	<0.0001 (+)
Use of D4T	0,0335 (+)
Use of NFV	<0.0001 (+)
Use of any NNRTI	0,0141 (+)
Time on RTV (full-dose) last scheme	0,0717
Time on RTV boosted PI last scheme	0,0001 (+)
Time on D4T last scheme	0,2575
Time on NFV last scheme	0,1744
Time on NNRTI last scheme	0,9131
**Genotype basal effect**	**0,1177** [1]
UTR 3238 (SsTI) CG vs CC	0,0092 (+)
UTR 3238 (SsTI) GG vs CC	0,2964
IRE -455 (FokI) CT vs TT	0,8904
IRE -455 (FokI) CC vs TT	0,9959
IRE -482 (MspI) TC vs CC	0,5887
IRE -482 (MspI) TT vs CC	0,9971
**Genotype treatment-associated effect**	**0,0003** [2]
Effect under RTV boosted PI shemes	
UTR 3238 (SsTI) CG vs CC	0,0001 (-)
UTR 3238(SsTI) GG vs CC	0,8641
IRE -482 (MspI) TC vs CC	0,4314
IRE -482 (MspI) TT vs CC	0,0063 (+)
Effect under D4T including shemes	
UTR 3238 (SsTI) CG vs CC	0,1243
UTR 3238(SsTI) GG vs CC	0,9566
IRE -455 (FokI) CT vs TT effect	0,7073
IRE -455 (FokI) CC vs TT effect	0,9948
IRE -482 (MspI) TC vs CC effect	0,2237
IRE -482 (MspI) TT vs CC effect	0,9910
**Genotype effect associated to time of exposure**	**0,0959** [3]
Interaction with accumulated HAART time	
IRE -455 (FokI) CT vs TT	0,4283
IRE -455 (FokI) CC vs TT	0,1542
Interaction with time on current scheme	
IRE -482 (MspI) TC vs CC	0,1132
IRE -482 (MspI) TT vs CC	0,5043

The contribution of each factor was evaluated with Wald test, p-values are depicted. The correlation sign is depicted between brackets. Test results for hierarchical model comparison are shown in bold (LRT).

[1] *vs Null hipothesis model*

[2] *vs Genotype basal effect model*

[3] *vs Genotype treatment-associated effect model*
