# Supplementary material for: Hypercholesterolemia Is Associated with the Apolipoprotein C-III (APOC3) Genotype in Children Receiving HAART: An Eight-Year Retrospective Study
Source: PLoS One. 2012 Jul 25;7(7):e39678. doi: 10.1371/journal.pone.0039678 (PMC3405089; doi:10.1371/journal.pone.0039678)
Supplement: Table S5 — Hierarchical test for absolute plasma levels difference under alternative LMM models for total cholesterol levels. (PDF) [file pone.0039678.s008.pdf]

Supplementary Table S5.Hierarchical test for absolute plasma levels difference under alternative LMM models for total cholesterol levels.

Model	Alt 3 vs. Null	Alt. 3 vs Alt 1	Alt. 3 vs Alt 2	Alt 2 vs. Null	Alt. 2 vs Alt 1	Alt. 1 vs Null
**Original model (table 4)**	<0.0001	<0.0001	0,0002	0,0008	0,0007	0,1672
**Allternative hypothesis regression terms without backward elimination (forced inclusion of all terms except 19 to 21, supplementary table 1)[2]**	<0.0001	<0.0001	0,0018	0,0006	0,0010	0,0956
**Alternative hypothesis without backward elimination with 4 drugs interaction terms (NNRTI and NFV in adition to D4T and RTV)[2]**	<0.0001	<0.0001	0,0878	<0.0001	<0.0001	0,0171
**BMI forced into original model[3]**	0,0002	0,0002	0,0330	0,0009	0,0007	0,1845
**CD4+ T cell counts and viral load forced into original model[4]**	<0.0001	<0.0001	0,0007	0,0005	0,0003	0,2086

Obseved significance on 127 patients (1556 determinations) are depicted (p values).

1Alt= Alternative hipothesis, Null= Null Hipothesis (Supplementary Figure S1)

[2] 3238 homozygotes (2 patients/25 determinations) excluded due to model estimation singularities

[3]n=111 patients (1445 determinations) due to missing data

[4]n=125 patients (1437 determinations) due to missing data
