# Supplementary material for: Hypercholesterolemia Is Associated with the Apolipoprotein C-III (APOC3) Genotype in Children Receiving HAART: An Eight-Year Retrospective Study
Source: PLoS One. 2012 Jul 25;7(7):e39678. doi: 10.1371/journal.pone.0039678 (PMC3405089; doi:10.1371/journal.pone.0039678)
Supplement: Algorithm S1 — Multiple fractional polynomials algorithm applied for model building. (PDF) [file pone.0039678.s009.pdf]

***Multiple fractional polynomials algorithm: flow diagram***

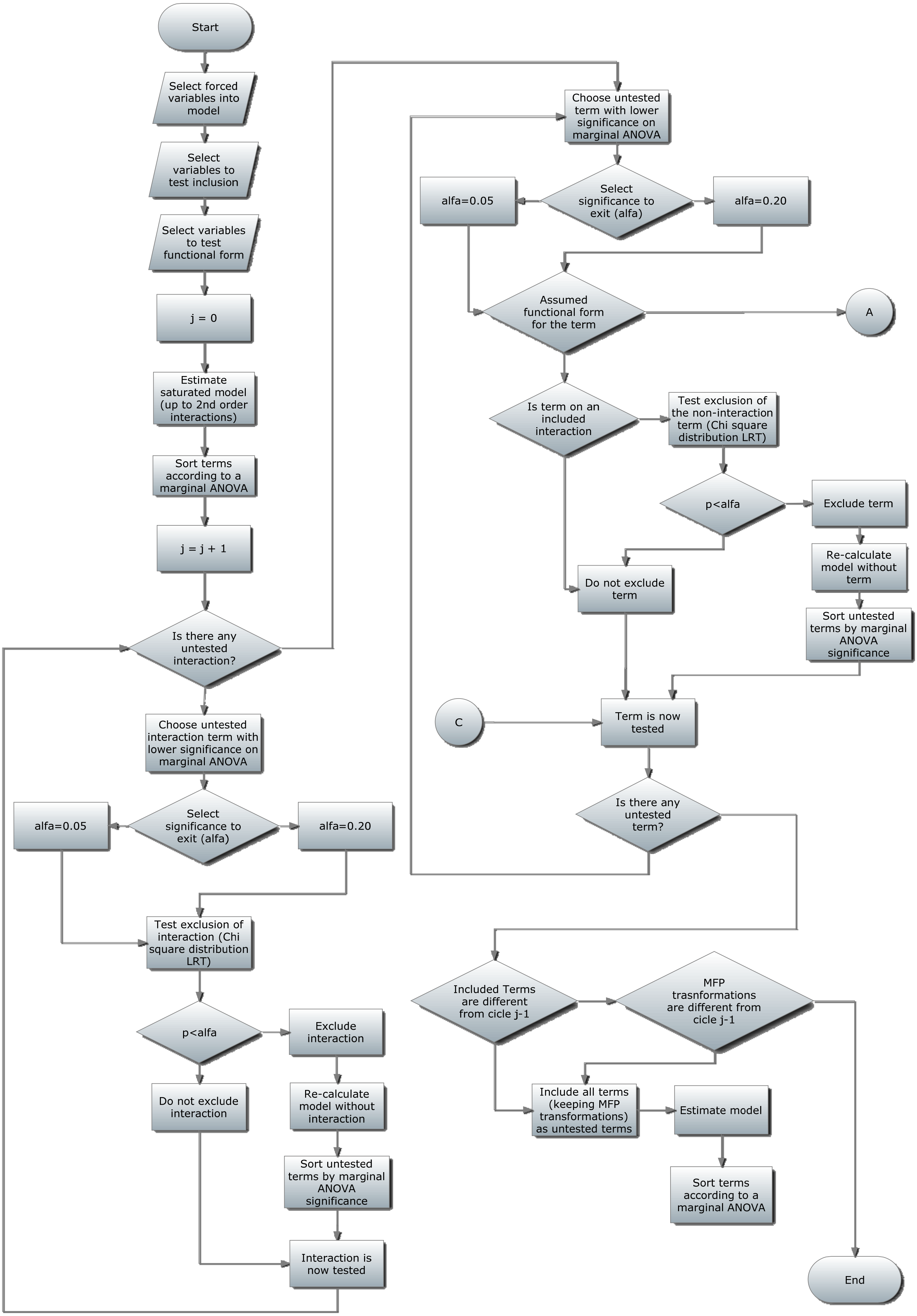
Start
Select forced variables into model
Select variables to test inclusion
Select variables to test functional form
j = 0
Estimate saturated model (up to 2nd order interactions)
Sort terms according to a marginal ANOVA
j = j + 1
Is there any untested interaction?
Choose untested interaction term with lower significance on marginal ANOVA
alfa=0.05
Select significance to exit (alfa)
alfa=0.20
Test exclusion of interaction (Chi square distribution LRT)
p<alfa
Exclude interaction
Do not exclude interaction
Re-calculate model without interaction
Sort untested terms by marginal ANOVA significance
Interaction is now tested
Choose untested term with lower significance on marginal ANOVA
alfa=0.05
Select significance to exit (alfa)
alfa=0.20
Assumed functional form for the term
A
Is term on an included interaction
Test exclusion of the non-interaction term (Chi square distribution LRT)
p<alfa
Exclude term
Re-calculate model without term
Do not exclude term
Sort untested terms by marginal ANOVA significance
C
Term is now tested
Is there any untested term?
Included Terms are different from cicle j-1
MFP trasnformations are different from cicle j-1
Include all terms (keeping MFP transformations) as untested terms
Estimate model
Sort terms according to a marginal ANOVA
End

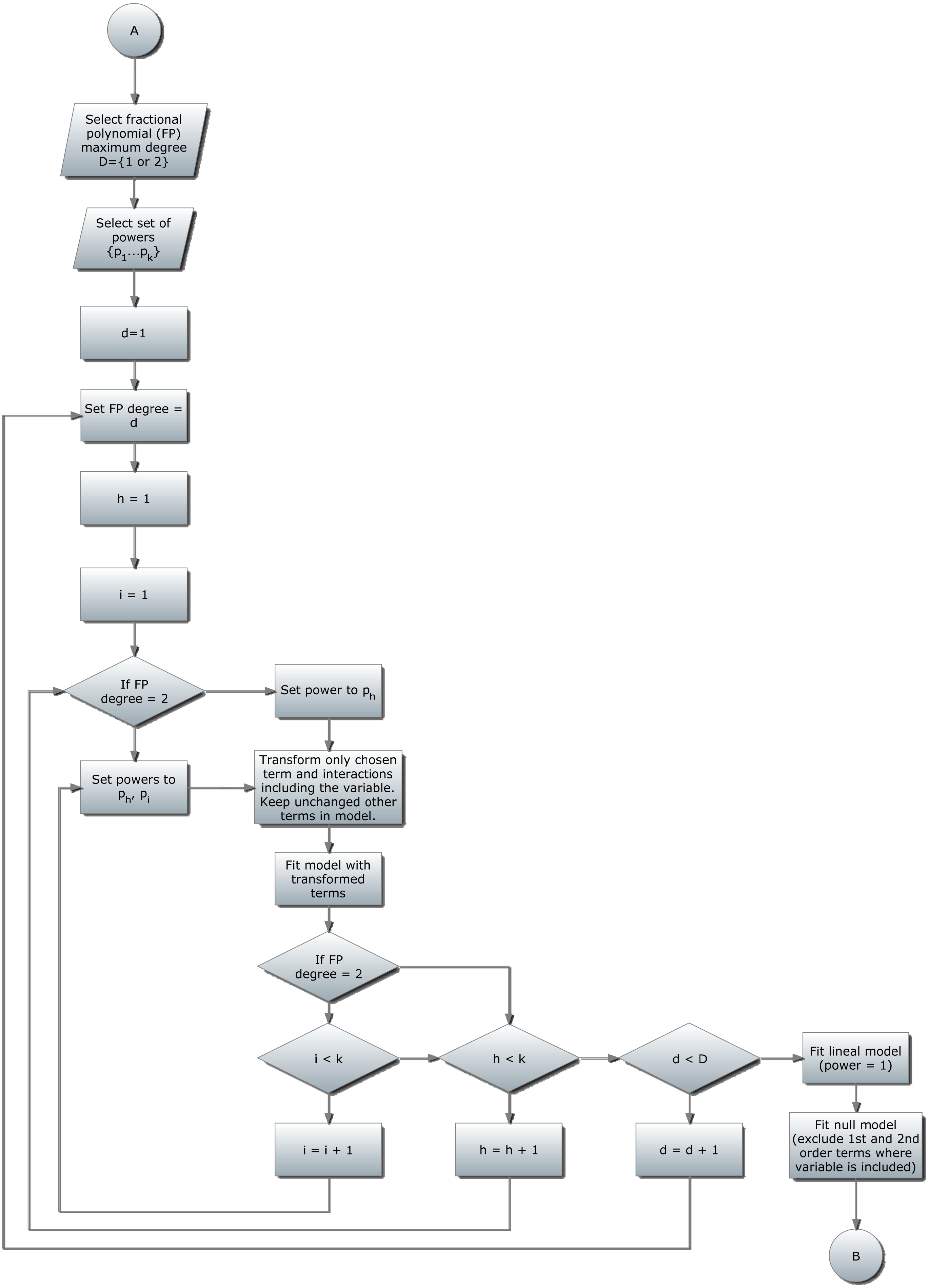

A
Select fractional polynomial (FP) maximum degree D={1 or 2}
Select set of powers {p1...pk}
d=1
Set FP degree = d
h = 1
i = 1
If FP degree = 2
Set power to ph
Set powers to ph, pi
Transform only chosen term and interactions including the variable. Keep unchanged other terms in model.
Fit model with transformed terms
If FP degree = 2
i < k
h < k
d < D
Fit lineal model (power = 1)
i = i + 1
h = h + 1
d = d + 1
Fit null model (exclude 1st and 2nd order terms where variable is included)
B

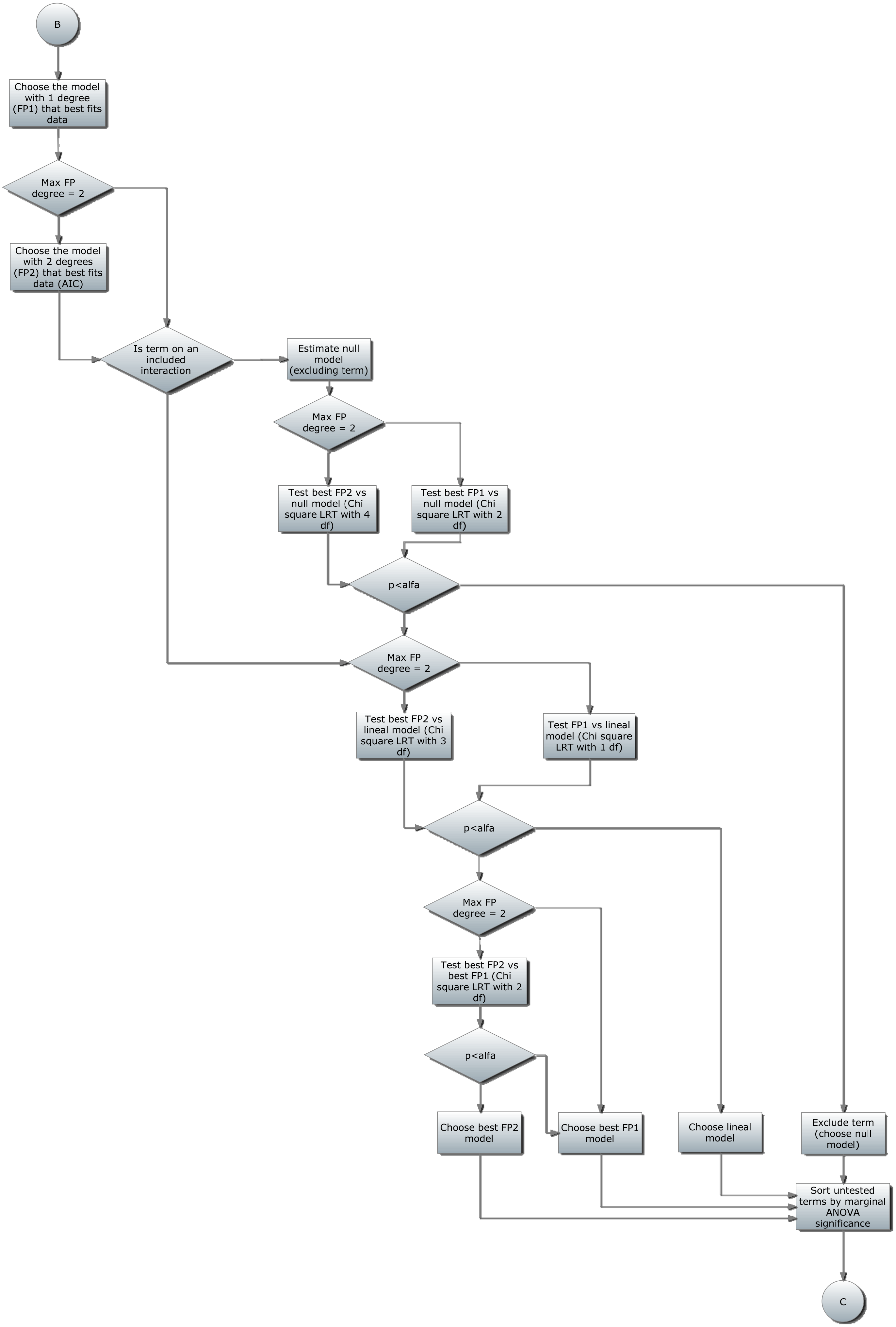
B
Choose the model with 1 degree (FP1) that best fits data
Max FP degree = 2
Choose the model with 2 degrees (FP2) that best fits data (AIC)
Is term on an included interaction
Estimate null model (excluding term)
Max FP degree = 2
Test best FP2 vs null model (Chi square LRT with 4 df)
Test best FP1 vs null model (Chi square LRT with 2 df)
p<alfa
Max FP degree = 2
Test best FP2 vs lineal model (Chi square LRT with 3 df)
Test FP1 vs lineal model (Chi square LRT with 1 df)
p<alfa
Max FP degree = 2
Test best FP2 vs best FP1 (Chi square LRT with 2 df)
p<alfa
Choose best FP2 model
Choose best FP1 model
Choose lineal model
Exclude term (choose null model)
Sort untested terms by marginal ANOVA significance
C

## *Multiple fractional polynomials algorithm: CRAN R script*

```
    library(nlme)

    ##Auxiliary functions (dont use on interactions)
    FP1<-function(termino,potencia){
    #termino=term to transform (string)
    #potencia = power
    termino.pp<-NULL
    termino.pp<-paste("I( (1+",termino," )^(",potencia,")  )") #the addition of 1 to avoid log transformation
of 0s, and 0 denominators
    for (pp in 1:length(potencia)){
            if (potencia[pp]==0) termino.pp[pp]<-paste("I(log(1+",termino,"))")
    }
    return(termino.pp)
    }

    FP2<-function(termino,potencia1,potencia2){
    #termino=term to transform (string)
    #potencia1 = power1
    #potencia2 = power2
    salida<-matrix(nrow=length(potencia1),ncol=length(potencia2))
    FPshort<-function(txx,p){
            if (p==0) short=paste("(log(1+",txx,"))") else short=paste("( (1+",txx," )^(",p,")  )")
            return(short)
            }
    FP2simple<-function(p1,p2,tx=termino){
            t1=FPshort(tx,p1)
            if (p1==p2) t2=paste("(",t1," *log(1+",tx,")",")") else t2=FPshort(tx,p2)
            return(paste("I",t1,"+I",t2))
            }
    for (i in 1:length(potencia1)) for (j in 1:length(potencia2)) salida[i,j]<-
FP2simple(potencia1[i],potencia2[j])
    dimnames(salida)<-list(paste("1erPot= ",potencia1),paste("2daPot= ",potencia2))
    return (salida)
    }


    #safeanova solves an inversion of the variable order in the interaction terms that appears with the use
of the anova or anova.lme functions.  This events  takes place occasionally, but also sistemastically with
some combinations of variables names. It might be derived from the manipulation of the variables names by
these funtions.
    safeanova<-function(modelolme, type,checkterms){
    #modelolme=lme object
    #type= type parameter to inherit to anova.lme
    #checkterms= list of string variables
            alltermsinmodel<-unlist(sapply(checkterms,function (x)
attr(terms(formula(paste("~",x))),"term.labels")) )
            names(alltermsinmodel)<-NULL
            anovabug<-anova.lme(object=modelolme, type=type)
            dimnames(anovabug)[[1]]<-c("(Intercept)",alltermsinmodel)
            return(anovabug)
    #returns anova.lme and checks if all terms are included in "checkterms"
    }


    takeout.MFP<-function(elmodelo,terminosMFPmod,checkterms){
    #function should be use when evaluating FP2. Avoids the grow of the list of anova terms -consequence of
estimating regression coefficients for two transformations.
    #elmodelo = lme object
    #terminosMFPmod = list. Each item contains matrix of strings (the names for all terms with
transformations)
            anova1<-safeanova(modelolme=elmodelo,type="marginal",checkterms=checkterms)[-1,]
            all.to.take.out<-NULL
            terminosmodelos<-nomes<-dimnames(anova1)[[1]]
            for (i in terminosMFPmod) if (!is.na(i[1])) {
                    for (j in 1:nrow(i)) {
                            anova1.i<-match(gsub(" ","",i[j,1]),gsub(" ","",terminosmodelos))
                            anova1.ii<-match(gsub(" ","",i[j,2]),gsub(" ","",terminosmodelos))
                            if (!is.na(anova1.i)){
                                    anova1[anova1.i,]<-
anova.lme(elmodelo,type="marginal",Terms=attr(terms(elmodelo),"term.labels")[c(anova1.i,anova1.ii)])
                                    nomes[anova1.i]<-paste(i[j,],collapse="+")
                            }#end IF the term exists
                            }#(j in nrow(i))
                    all.to.take.out<-c(all.to.take.out,gsub(" ","",i[,2]))
                    }#(i in terminosMFPmod)
            dimnames(anova1)[[1]]<-nomes
            all.to.take.out<-match(all.to.take.out,gsub(" ","",terminosmodelos))
            all.to.take.out<-all.to.take.out[!is.na(all.to.take.out)]
            anova1<-anova1[-all.to.take.out,]
            return(list(anovaMFP=anova1,terminos.MFP.repetidos=all.to.take.out))
                    #Returns:
                            #$anovaMFP: corrected anova: FP2 derived terms are evaluated jointly
```

```
                            #$terminos.MFP.repetidos: excluded terms from the anova function ("anova1") to
result in $anovaMFP
            }



    #####END auxiliary functions


    ####MFP.lme: Fractional polynomial (FP) algorithm for model building and backward elimination
    ####nulo: Null model. Null model variables are not tested to be included. Formula should include
response: "resp ~ var1 + var2 +..."
    ####fixed: list of variables to test exclusion with p<alfa
    ####confundentes: (counfounders) list of variables to test exclusion with p<alfa.conf
                    #How does program react to duplicated term? -> takes out fixed terms already included in
nulo, takes out confounders already included in nulo or fixed
    ####MFPon (multiple variables FP) : list of continous variables (should not have negative value
observations) to test functional alternatives to lineal. Term exclusion is tested with corresponding p value
(fixed or counfounders). If term is included in nulo, it will not be excluded in any case.
            ####terms for MFP should be included (in the linear form) in "nulo", "fixed" or "confundentes"
!!!!
    ####random: random terms for lme model

            #fixed, confundentes, random should be specified as formulas (only right side):  "~ var1 + var2 +
var*var3 +..."
    #####NOTE: FP tests employ: alfa for "fixed" and alfa.conf for "confundentes" (for all steps in FP
algorithm)
    ####int.drive= how to handle tests for interactions. TRUE (default) means that:
    #if interaction is significative then interaction term (second order term), and the two first order terms
involving variables included in the interaction are included in (i.e. not excluded from) model (without
further testing)
    ####int.drive=FALSE: in this case, first order terms are tested at the same time than interaction term:
marginal anova is carried out for the joint exclusion of 2nd order term and the two first order terms. The
test takes place when evaluating interaction, and no further testing is performed later on the cycle

    ####Aditional parameters:
            #MFPpot: set of power transformations to test. (see Royston, 1999)
            #FPdegree: max degree for FP (only 1 or 2 supported)
            #method, na.action, correlation, data, subset: parameters to be inherited to lme fuciton (nlme)
                    #(method should always be "ML")
            #historial: TRUE keeps track of all lme models estimated during iteration

    MFP.lme<-function(nulo,fixed,random,confundentes,MFPon,MFPpot=c(-1,-
0.5,0,0.5,1,2),FPdegree=2,method="ML",na.action="na.omit",correlation=NULL,data,subset=NULL,alfa=0.05,alfa.co
nf=0.2,int.drive=FALSE,historial=FALSE){
    print(as.name("--------- Multiple Fractional Polynomials (MFP) for lme v1.17 ---------"))
    caller<-match.call()
    #terms handling
    nuloterminos<-attr(terms(nulo),"term.labels")
    fixterminos<-attr(terms(fixed),"term.labels")
    confterminos<-attr(terms(confundentes),"term.labels")
    mfpterminos<-attr(terms(MFPon),"term.labels")
    response<-all.vars(nulo)[1]

    #take out duplicated terms
    sacarfix<-match(nuloterminos,fixterminos,0)
    sacarconf<-unique(c(match(nuloterminos,confterminos,0),match(fixterminos,confterminos,0)))
    sacarfix<-switch(1+(sum(sacarfix) >0),1:length(fixterminos), -sacarfix)
    sacarconf<-switch(1+(sum(sacarconf)>0),1:length(confterminos),-sacarconf)
    if (length(fixterminos)>0) fixterminos<-fixterminos  [sacarfix]
    if (length(confterminos)>0) confterminos<-confterminos[sacarconf]
    allterms<-
attr(terms(eval(formula(paste(response,"~",paste(c(nuloterminos,fixterminos,confterminos),collapse=" +
"))))),"term.labels")
    alltermsFIJO<-allterms ###alltermsFIJO is not further modified; in contrast to "allterms" that changes
with MFP
    alltermsorder<-
attr(terms(eval(formula(paste(response,"~",paste(c(nuloterminos,fixterminos,confterminos),collapse=" +
"))))),"order")
            #the order is later used to distinguish two way interactions (2nd order) from 1st order terms.
2nd order terms are tested first


    ####bug fix v1.15
    ####terms names (character strings) are corrected to avoid intermittent inconsistencies (?) when
comparing allterms and nulo,fixed,confundentes (index.terms)

    nuloterminos<-allterms[0:length(nuloterminos)]

    ####bug fix v1.16
    if (length(fixterminos)>0) fixterminos<-
allterms[(length(nuloterminos)+1):(length(nuloterminos)+length(fixterminos))]
    if (length(confterminos)>0) confterminos<-
allterms[(length(nuloterminos)+length(fixterminos)+1):length(allterms)]
```

```
    ####

    #términos index
    index.terms<-list(
    nulo=sapply(allterms ,function(x,y) sum(x==y)>0,nuloterminos),
    fixed=sapply(allterms ,function(x,y) sum(x==y)>0,fixterminos),
    confundentes=sapply(allterms ,function(x,y) sum(x==y)>0,confterminos),
    mfp=sapply(allterms ,function(x,y) sum(x==y)>0,mfpterminos),
    tested=rep(FALSE,length(allterms)),
    include=rep(TRUE,length(allterms))
    )
    names(index.terms$include)<-names(index.terms$tested)<-allterms
    #term names in index and allterms have the same arrangement, that is constant during run. No
modifications should be introduced except for MFP terms
    #index.tested.include change aftert testing each term (resets each cycle)

    ###saturated lme
    formlme<-formula(paste(response,"~",paste(allterms,collapse=" + "))) #formula saturada
    lmepar<-c("data","random","correlation","subset")
    parlme<-match(lmepar,names(caller))
    calllme<-caller[c(1,parlme)]
    calllme[1]<-call("lme")
    calllme$fixed=formlme
    calllme$na.action=na.action
    calllme$method=method
    calllme$keep.data=FALSE
    mod.sat.lin<-eval(calllme)

    #terms sorting
    #order 1st
    #p-value 2nd
    rank.sat.lin<-sort.list(anova(mod.sat.lin,type="marginal")[-1,4], decreasing =
TRUE,method="quick",na.last=NA)#p-value...
    rank.sat.order<-attr(terms(mod.sat.lin),"order")[rank.sat.lin]#orden por signif.
    rank2<-sort.list(rank.sat.order, decreasing = TRUE,method="radix")#orden por orden de factores
    rank.sat.lin<-rank.sat.lin[rank2]
    rank.sat.order<-rank.sat.order[rank2]

    ########## MFP, definitions and controls ############
    if (length(MFPpot)==0) {
            MFPpot<-c(-1,-0.5,0,0.5,1,2)
            warning("Power set is not valid. Using default set {-1,-0.5,0,0.5,1,2}")
            }
    if (any(alltermsorder[index.terms$mfp]!=1)){
            print(as.name("Terms in MFP should always be 1st order (no interactions allowed)"))
            warning("2nd order terms were excluded from MFP evaluation")
            index.terms$mfp[alltermsorder!=1]<-FALSE
            }
    if (any(is.na(match(mfpterminos,allterms)))) {print(as.name("MFP terms should also be included as null
model, fixed or counfounding terms"))
            warning("Some terms were not MFP evaluated because of model omision")
            }

    ##check if the names of variables to MFP are included in the character string of other variables names
(avoid grep function error)
    mfpterminos.ctrl<-mfpterminos
    mfp.passed=0
    if (length(mfpterminos)>0) while (mfp.passed==0){
            mfp.passed=1
            for (controlMFP in 1:length(mfpterminos)){
                    if (length(grep(gsub(" ","",mfpterminos.ctrl[controlMFP]),gsub("
","",allterms[alltermsorder==1])))>1){
                            mfpterminos[controlMFP]<-allterms[match(mfpterminos[controlMFP],allterms)]<-
paste(mfpterminos[controlMFP],"@@",sep="")
                            mfp.passed=0
                            }#if mfpterminos in allterms
                    }#for controlMFP
            }#while mfp.passed==0

    MFP.p0<-matrix(rep(c(1,NA),length(allterms)),ncol=length(allterms)) #all variables start linear
    dimnames(MFP.p0)<-list(c("p1","p2"),alltermsFIJO)
    MFP.p1<-MFP.p0 #MFP at cycle end
    #bug fix V1.16,  before: "modelsofarMFP<-list()"
    modelsofarMFP<-list(NA) #modelsofarMFP the list includes all MFP terms that needs transformations (NA)
                                    #but only fills FP2 transformations (that estimates two regression
coefficients)
    if (length(mfpterminos)>0) for (i in 1:length(mfpterminos)) modelsofarMFP[[i]]<-NA

    names(modelsofarMFP)<-alltermsFIJO[index.terms$mfp]


    ####cycle
    convergencia=0#convergence condition: convergencia=1
    alltermsold<-allterms #save past cycle data
```

```
            #NOTE: allterms includes selected FP transformations
    modold<-mod.sat.lin#modold: lme model from previous cycle
    modvar<-mod.sat.lin#modvar: lme model from previous variable (same cycle)
    varanova<-safeanova(modvar,type="marginal",checkterms=allterms)[-1,]
    include.OLD<-index.terms$include #variables included in model (all of them at the begining)
    contador.ciclo<-0  #cycle count
    proxrank<-rank.sat.lin
    proxorder<-rank.sat.order

    #iteration history
    Historico<-list()
    if (historial) {
            Historico<-list(incluidas=matrix(rep(TRUE,length(allterms)),ncol=length(allterms)), MFP=
list(MFP.p1[,index.terms$mfp]), Anova=list(varanova), ranking.progresivo=list())
            Historico$ranking.progresivo[[1]]<-list(Inicio=alltermsFIJO[proxrank])
            dimnames(Historico$incluidas)[[2]]<-alltermsFIJO
            rankprogesiv<-list()
            }
            ## For each cycle
            ## MFP and final anova results included
            ## "ranking.progresivo" keps track of variable ranking changes during cycle -while testing each
variable-
            ## start cycle 0 (saturated model - always linear)



    ##################BACKWARD ELIMINATION PROCEDURE + MFP#####################################
    ##################variable selection and best FP transformation#####################################
    ##################Each cycle starts with all initial variables, but FP are inherited from previous cycle
    ##################Convergence conditions are fullfilled when FP transformations and variables
excluded/included are kept from previous cycle

    while (convergencia==0){
    #####during each cycle every term is tested again
    #####convergence if MFP.p0==MFP.p1 and include.OLD=index.terms.include

    variable=0
    contador.ciclo=contador.ciclo+1
    print(as.name("-----------------------------------------------------"))
    print(as.name(paste("Cycle...",contador.ciclo,"...")))
    MFP.p0<-MFP.p1 #initial MFP is end MFP
    modold<-modvar #old model is actual model
    include.OLD<-index.terms$include
    #reset all variables to "untested"
    index.terms$tested<-rep(FALSE,length(allterms))
    index.terms$tested[index.terms$nulo]<-TRUE
    index.terms$include=rep(TRUE,length(allterms))

    if (contador.ciclo>1){
    ##model restart#################################################################
            ##restart everything except MFP transformations (included in allterms)
            calllmenow<-modvar$call
            formlme<-formula(paste(response,"~",paste(allterms,collapse=" + "))) #refresh formula (+MFP
transformations)
            calllmenow$fixed=formlme
            modvar<-eval(calllmenow)
                    #sort variables again
                    #same criteria: term order 1st, significance 2nd

            ##correction to test FP2 transformed terms in one step, since terms are duplicated (1 coefficient
estimation for each power transformation)
            if (any(!is.na(unlist(modelsofarMFP)))){
                    nueva.anova<-
takeout.MFP(elmodelo=modvar,terminosMFPmod=modelsofarMFP,checkterms=allterms[index.terms$include])
                    varanova<-nueva.anova$anovaMFP
                    incluir.términos<-(1:length(attr(terms(modvar),"order")))[-
nueva.anova$terminos.MFP.repetidos]
                    } else {
                    varanova<-safeanova(modvar,type="marginal",checkterms=allterms[index.terms$include])[-1,]
                    incluir.términos<-(1:length(attr(terms(modvar),"order")))
                    }
            ########## sort varanova again ##########
            var.rank.closed<-sort.list(varanova[,4], decreasing = TRUE,method="quick",na.last=NA)
            rank.sat.order<-(attr(terms(modvar),"order")[incluir.términos])[var.rank.closed]  #take out
modvar terms already excluded in varanova
            rank2.closed<-sort.list(rank.sat.order, decreasing = TRUE,method="radix")
            var.rank.closed<-var.rank.closed[rank2.closed] #1st sorting criteria
            rank.sat.order<-rank.sat.order[rank2.closed] #2nd sorting criteria

            ##allterms should match the row of varanova:
            alltermscollapsed<-sapply(allterms,function (x)
paste(attr(terms(formula(paste("~",x))),"term.labels"),collapse="+")) #alltermscollapsed: collapsed
interaction terms
```

```
            var.rank.closed<-match(gsub(" ","",dimnames(varanova)[[1]]),gsub("
",""),alltermscollapsed))[var.rank.closed]   #match varanova to allterms,

            rank.out<-match(proxrank[1:variable],var.rank.closed,nomatch=0)
            rank.out<-switch(rank.out==0,1:length(var.rank.closed),-rank.out)

            proxrank<-c(proxrank[1:variable],var.rank.closed[rank.out])
            proxorder<-c(proxorder[1:variable],rank.sat.order[rank.out])
    } #end of model restart
    ##################################

    #Some variations resulting from each variable test: For variable i being test (from 1 to I),
    #proxrank: sort again variables i+1 to I (no change for variables 1 to i, which are already tested)
    #proxorder: idem
    #varanova: new tests, plus FP2 correction to collapse transformations into 1 term
    #allterms: keeps variable order (=alltermsFIJO), but FP transformations are included
    #alltermsFIJO: constant (this is useful to choose linear term)
    #index.terms: tested variables and included variables are refreshed every time
    #modvar: lme model excluds tested variables up to variable i that had p>alfa
    #calllme: lme call terms are updated before generating modvar. Likewise, callMFP is updated to use in MFP




    #################################
    #################################
            while (variable < length(proxrank)){                    #NOTA: proxrank length is constant: FP2
duplicates the number of MFP terms, but this is corrected at the end of each cycle
                    variable<- variable +1
                    varranknext<-proxrank[variable]                 #rank for the next variable to test (list
order keeps intact)

                    print(as.name(paste("Evaluating inclusion of the regression term -
",allterms[varranknext],"-  [cycle ",contador.ciclo,"variable ",variable,"]")))

                    if
(((index.terms$fixed+index.terms$confundentes)*(!index.terms$mfp)*(!index.terms$tested))[varranknext]) {


                            if (proxorder[variable]>1){ ##(i.e. ==2)
                                            variables.i<-
paste((attr(terms(as.formula(paste("~",alltermsFIJO[varranknext]))),"variables")))[-1]
                                            v1.interact<-match(gsub(" ","", variables.i[1] ),gsub(" ","",
alltermsFIJO[index.terms$include]))
                                            v2.interact<-match(gsub(" ","", variables.i[2] ),gsub(" ","",
alltermsFIJO[index.terms$include]))
                                            v12.interaction<-match(gsub(" ","", allterms[varranknext]),gsub("
",""), allterms[index.terms$include]))
                                            }# este bloque es de utilidad para los algoritmos 1 y 2 en
distintos momentos

                            #(

                            ######2 alternative algorithms for the test of interactions (int.drive):
                            ############################################################
                            ############################################################
                            ##### Algorithm 1...
                                            #test 2nd order term.
                                            #1st order terms are not excluded if 2nd order term is not
excluded (2nd order terms are always tested before). Thus, if interaction is included 1st order terms are not
tested (2nd order inclusion sets $tested=TRUE for the 2 1st order terms involved)
                                            #if interaction is excluded, first order terms are tested
independently
                            if ((int.drive==TRUE)|(proxorder[variable]==1))
index.terms$include[varranknext]<-varanova[
                                            match(gsub(" ","",

paste(attr(terms(formula(paste("~",allterms[varranknext]))),"term.labels"),collapse="+"))
                                                    ,gsub(" ","",dimnames(varanova)[[1]])),4] <

(as.integer(index.terms$fixed)*alfa+as.integer(index.terms$confundentes)*alfa.conf)[varranknext] #test
variable
                                                                                                    #gsub corrects
some inconsistencies in the terms names

                            ##### Algorithm 2...
                                            #at the time of testing 2nd order terms: jointly test interaction
term + the two 1st order terms
                                            #if the test of the interaction+two 1st order terms has p<alfa
then interaction and 1st order terms are not excluded (actually, as algorithm 1)
                                            ##if interaction is excluded, first order terms are tested (one
at the time) independently

                            if ((int.drive==FALSE)&(proxorder[variable]>1)){
```

```
                                                                                          call.minus<-
calllme
    
call.minus$fixed<-formula(paste(response,"~",paste((allterms[index.terms$include])[-
c(v12.interaction,v1.interact,v2.interact)],collapse=" + ")))
                                                                                          modvar.minus<-
eval( call.minus)

index.terms$include[varranknext]<-anova.lme(modvar,modvar.minus)$"p-value"[2] <

(as.integer(index.terms$fixed)*alfa+as.integer(index.terms$confundentes)*alfa.conf)[varranknext] #test
variable

                            }       #int.drive==FALSE

                            #####decisions:
                            if (!index.terms$include[varranknext]) {
                                    #case: variable IS excluded
                                    #
                                    calllme$fixed<-
formula(paste(response,"~",paste(allterms[index.terms$include],collapse=" + ")))
                                    modvar<-eval(calllme)
                                    ##careful!! modvar terms are mixed due to MFP


                                    #end of case: variable IS excluded ($include= false)
                            } else{
                                    #case: variable IS NOT excluded
                                    #
                                    if (proxorder[variable]>1) {            #2nd order inclusion -> 1st order
inclusion (x2)
                                            print(as.name(paste("Interaction' 1st order terms are

included",allterms[varranknext])))
                                            index.terms$tested[match(gsub(" ","",variables.i),gsub("

","",allterms))]<-TRUE
                                            }

                            } #end of case: variable IS NOT excluded


            } #end if (fix or conf)

            if (index.terms$mfp[varranknext]) { #do not look $tested (so "nulo" terms are tested)

            print(as.name("Evaluating Fractional Polynomials (FP)"))
            callMFP<- modvar$call

            termsfornull<-termsforlinear<-index.terms$include #to discard excluded variables (up to variable
i-1)
            terminosMFP<-grep(allterms[varranknext],allterms,fixed=TRUE) #serch regression terms that include
variable
            ##null model will include terms where index.terms$inlude=TRUE (and do not include MFP variable)
            if (length(     terminosMFP)>0) termsfornull[terminosMFP]       <-FALSE


            ##############################################################################
            ###############-------Multiple Fractional Polynomials----------###############
    #W. Sauerbrei and P. Royston (1999): Backwards elimination procedure for multiple variables
    #G. Ambler and P. Roystin (2001) : RA2
    ##
    ##      1) Choose p-value                               ---Test significance level
    ##      2) Test FP2 vs null (chisq 4 d.f.)              ---Overall
    ##      3) Test FP2 vs linear (chisq 3 d.f.)            ---No-linearity test
    ##      4) Test FP2 vs FP1 (chisq 2 d.f.)               ---2nd degree
    ##
    ## omit 2) if linearity is assumed
    ##########################################################################################################
#####
    #######
    #
    #####Model generation

    #Linear model
    alltermsHIBRIDO<-
replace(allterms,(1:length(allterms))[terminosMFP],gsub(alltermsFIJO[varranknext],paste("I(1+",alltermsFIJO[v
arranknext],")"),alltermsFIJO,fixed=TRUE)[terminosMFP])
            #Important note: despite the significance of a FP transformation for the variable being evaluated
is tested against its linear form, the remaining terms should keep the latest FP transformations derived from
the cycle
            #"1+..." is needed to cope with variables with 0s (when power is 0 -logarithmic transformation-
or negative)

    formMFP<-formula(paste(response,"~",paste(alltermsHIBRIDO[termsforlinear],collapse=" + ")))
    callMFP$fixed<-formMFP
```

```
    modelo.p1 <-eval(callMFP)

    #Null model
    lin.vs.null<-NA
    if (index.terms$nulo[varranknext]) modelo.null<-modelo.p1 else {
            formMFP<-formula(paste(response,"~",paste(allterms[termsfornull],collapse=" + ")))
            callMFP$fixed<-formMFP
            modelo.null<-eval(callMFP)
            lin.vs.null<-anova(modelo.null,modelo.p1)$"p-value"[2]
            }

    #####FP2#####
    if (FPdegree>1){

            FP2.factors<-FP2(alltermsFIJO[varranknext],MFPpot,MFPpot)
                            ####Note: FP2.factors[j,i]==FP2.factors[i,j]  (in term names variables have
inverted order)
            ####FP2.factors: allterms + power...
            alltermsFP2<-list()
            for (pi in 1:length(FP2.factors)) alltermsFP2[[pi]]<-
gsub(allterms[varranknext],paste("(",FP2.factors[pi],")"),allterms,fixed=TRUE)
            names(alltermsFP2)<-c(outer(MFPpot,MFPpot,function (x,y) paste("p1=",x," p2=",y,sep="")))
            FP2.potencias<-cbind(p1=rep(MFPpot,times=length(MFPpot)),p2=rep(MFPpot,each=length(MFPpot)))
            ####Duplications are eliminated
            sacar.repetidos<-matrix(rep(TRUE,length(alltermsFP2)),ncol=ncol(FP2.factors))
            for (i in 1:(nrow(sacar.repetidos)-1)) for (j in (i+1):nrow(sacar.repetidos))
sacar.repetidos[i,j]<-FALSE
            alltermsFP2<-alltermsFP2[c(sacar.repetidos)]
            FP2.potencias<-FP2.potencias[c(sacar.repetidos),]
                    #Note: nº of formulas= (n^2+n)/2; n=length(MFPpot)

            ####Tests results...
            testFP2<-
rbind(nulo=c(NA,NA,modelo.null$logLik,summary(modelo.null)$AIC,1*index.terms$nulo[varranknext],NA,NA),lineal=
c(NA,NA,modelo.p1$logLik,summary(modelo.p1)$AIC,1,lin.vs.null,NA))
            dimnames(testFP2)[[2]]<-c("p1","p2","logLik","AIC","df","p-val/Ho:null","p-val/Ho:linear")
            print(as.name(paste("Building FP2 models for ",alltermsFIJO[varranknext],"(power
set:",paste(MFPpot,collapse=", ") ,")")))
            for (armamodel in 1:length(alltermsFP2)){
                    callMFP$fixed<-
formula(paste(response,"~",paste((alltermsFP2[[armamodel]])[index.terms$include],collapse=" + ")))
                    modelo<-eval(callMFP)
                    FPgl<-length(modelo$coef$fixed)-length(modelo.null$coef$fixed)+2 ##adds 2 df (2 exponents
for FP)
                    FPlogLik<-modelo$logLik
                    FPAIC<-summary(modelo)$AIC
                    FPp0<-1-pchisq(2*(modelo$logLik-modelo.null$logLik),FPgl)
                    FPp1<-1-pchisq(2*(modelo$logLik-modelo.p1$logLik),FPgl-1)
                    testFP2<-rbind(testFP2,c(FP2.potencias[armamodel ,],FPlogLik,FPAIC,FPgl,FPp0,FPp1))
                    }
            dimnames(testFP2)[[1]]<-c("nulo","lineal",names(alltermsFP2))

            } #FP2



    #####FP1#####
    MFPpot.sin1<-MFPpot[MFPpot!=1] #saco potencia 1, ya que siempre tengo que hacer el modelo
    FP1.factors<-FP1(termino=alltermsFIJO[varranknext],potencia=MFPpot.sin1)
    alltermsFP1<-list()
    for (pi in 1:length(FP1.factors)) alltermsFP1[[pi]]<-
gsub(allterms[varranknext],FP1.factors[pi],allterms,fixed=TRUE)
    names(alltermsFP1)<- paste("p1=",MFPpot.sin1,sep="")
    print(as.name(paste("Building FP1 models for ",alltermsFIJO[varranknext],"(power
set:",paste(MFPpot,collapse=", ") ,")")))

    ####resultados de los tests...
    testFP1<-
rbind(nulo=c(NA,NA,modelo.null$logLik,summary(modelo.null)$AIC,1*index.terms$nulo[varranknext],NA,NA),lineal=
c(1,NA,modelo.p1$logLik,summary(modelo.p1)$AIC,1,lin.vs.null,NA))
    dimnames(testFP1)[[2]]<-c("p1","p2","logLik","AIC","df","p-val/Ho:null","p-val/Ho:linear")

    for (armamodel in 1:length(alltermsFP1)){
            callMFP$fixed<-
formula(paste(response,"~",paste((alltermsFP1[[armamodel]])[index.terms$include],collapse=" + ")))
            modelo<-eval(callMFP)
            FPgl<-length(modelo$coef$fixed)-length(modelo.null$coef$fixed)+1 ##adds 1 df (1 exponent for FP)
            FPlogLik<-modelo$logLik
            FPAIC<-summary(modelo)$AIC
            FPp0<-1-pchisq(2*(modelo$logLik-modelo.null$logLik),FPgl)
            FPp1<-1-pchisq(2*(modelo$logLik-modelo.p1$logLik),FPgl-1)
            testFP1<-rbind(testFP1,c(MFPpot.sin1[armamodel],NA,FPlogLik,FPAIC,FPgl,FPp0,FPp1))
            }
    dimnames(testFP1)[[1]]<-c("nulo","lineal",names(alltermsFP1))
```

```
    ###############################################
    # 1) Test level
            alfa.to.exit<-
(as.integer(!index.terms$confundentes)*alfa+as.integer(index.terms$confundentes)*alfa.conf)[varranknext]
    ### D.F. calculation:
    ############  ->2x (FP degree)
    ############  ->1 for linear
    ############  ->0 for null
    ############ for interactions:
    ############ => d.f.= number of regression terms (including MFP variable) + (FP degrees: 1 or 2)

    # 2) Overall
    ##
            #AIC to choose best FP model

            bestFP1<-(1:nrow(testFP1))[testFP1[,4]==min(testFP1[-(1:2),4])]
            overall<-testFP1[bestFP1,]
            if (FPdegree>1){
                    bestFP2<-(1:nrow(testFP2))[testFP2[,4]==min(testFP2[-(1:2),4])]
                    if (testFP2[bestFP2,4]<testFP1[bestFP1,4]) overall<-testFP2[bestFP2,]
                    }
            overall.test<-overall[6]<alfa.to.exit
                                    ##Note: do not need this test if variable is included in null model or in
an included interaction. Done it anyways

    # 3) Non-linearity
    ##
            lineal.test<-overall[7]<alfa.to.exit

    # 4) FP- 2nd degree vs 1st
            FP2.test<-FALSE
            if (FPdegree>1) FP2.test<-1-pchisq(2*(testFP2[bestFP2,3]-
testFP1[bestFP1,3]),df=(testFP2[bestFP2,5]-testFP1[bestFP1,5])+1)<alfa.to.exit
            MFP.p1[,varranknext]<-overall[1:2]

    ###############################################

    ##############
    #Test were done, consequences are:
    #Exclusions/Inclusions...
            #Very Important Note!!
            #      excluded interactions remained excluded for MFP algorithm
            #      if FP2 vs linear IS rejected (and so variable should be included, at least in linear form)
then, variable is transformed in every 1st and 2nd order terms that were not previously excluded (despite 2nd
order terms were already tested)
            #      BUT if FP2 vs linear IS NOT rejected, all terms (1st and 2nd order) involving the variable
are excluded. Thus, 2nd order terms are now excluded, even when they were not before

    ##Variable exclusion
    if (!overall.test){
            allterms[terminosMFP]<-alltermsFIJO[terminosMFP] #back to linear form
            index.terms$include[terminosMFP]<-FALSE
            index.terms$include[index.terms$nulo]<-TRUE #null model terms
            if (sum(index.terms$nulo[terminosMFP])<length(terminosMFP)){ #if all terms are in the null model,
the linear model is not modified
                    ##take out variable (1st & 2nd order terms)...
                    if (sum(index.terms$nulo[terminosMFP])==0) modvar<-modelo.null
                    if (sum(index.terms$nulo[terminosMFP])>0){
                            formMFP<-
formula(paste(response,"~",paste(allterms[index.terms$include],collapse=" + ")))
                            callMFP$fixed<-formMFP
                            modvar<-eval(callMFP)
    ##warning: modvar order is modified due to FP2' duplication of terms

                            }#sum(index.terms$nulo)>0


                    #end if sum(index.terms$nulo[terminosMFP])<length(terminosMFP)
                    } else{         #sum(index.terms$nulo[terminosMFP])==length(terminosMFP)
                                    modvar<-modelo.p1
                                    allterms<-alltermsHIBRIDO
                    }#sum(index.terms$nulo)==length(terminosMFP)

            modelsofarMFP[[match(gsub(" ","",alltermsFIJO[varranknext]),gsub("
","",names(modelsofarMFP)))]]<-NA

            }#!overall

    ##Linear is best fit... =>
    if ((overall.test)&(!lineal.test)){
    #modvar queda lineal
```

```
            modvar<-modelo.p1
            modelsofarMFP[[match(gsub(" ","",alltermsFIJO[varranknext]),gsub("
","",names(modelsofarMFP)))]]<-NA
            allterms<-alltermsHIBRIDO
            MFP.p1[,varranknext]<-c(1,NA)

    } #end if linear


    #non-linearity evidence...=>
    if ((overall.test)&(lineal.test)){ ##might be FP1 or FP2:

            if (FP2.test) { #Test FP1 vs FP2
                    allterms<-alltermsFP2[[bestFP2-2]]
                    callMFP$fixed<-formula(paste(response,"~",paste(allterms[index.terms$include],collapse="
+ ")))
                    MFP.p1[,varranknext]<-testFP2[bestFP2,1:2]

                    #identify added/modified terms in modvar
                    ffMFP<-formula(paste(response,"~",paste(allterms,collapse=" + ")))            #all terms
are identified, even if they are not in current model
                    nuevosTerminosMFP<-
grep(alltermsFIJO[varranknext],attr(terms(ffMFP),"term.labels"),fixed=TRUE)
                    terms1<-nuevosTerminosMFP[(1:(length(nuevosTerminosMFP)/2))*2-1]
                    terms2<-nuevosTerminosMFP[(1:(length(nuevosTerminosMFP)/2))*2]  # =term1+1
                    modelsofarMFP[[match(gsub(" ","",alltermsFIJO[varranknext]),gsub("
","",names(modelsofarMFP)))]]<-
cbind(attr(terms(ffMFP),"term.labels")[terms1],attr(terms(ffMFP),"term.labels")[terms2])

                                    #modelsofarMFP: term names (strings) that include the MFP variable, only
in the case of FP2. format: list, each element is a two-columns matrix

                    }else{          #end FP2
                    allterms<-alltermsFP1[[bestFP1-2]]
                    callMFP$fixed<-formula(paste(response,"~",paste(allterms[index.terms$include],collapse="
+ ")))
                    MFP.p1[,varranknext]<-testFP1[bestFP1,1:2]
                    modelsofarMFP[[match(gsub(" ","",alltermsFIJO[varranknext]),gsub("
","",names(modelsofarMFP)))]]<-NA
                    }               #end FP1
            modvar<-eval(callMFP)

    ##warning: modvar terms might be mixed


            }#FP==TRUE

            }#if mfp



    ##############################################################################################################
    ##############################################################################################################
    ##############################################################################################################
    #######For every variable:
    ##Before finishing testing...
    ########################################################

            index.terms$test[varranknext]<-TRUE

    ########## re-make varanova ##########
            ##extra terms due to FP2 are going to be corrected...
            if (any(!is.na(unlist(modelsofarMFP)))){
                    nueva.anova<-
takeout.MFP(elmodelo=modvar,terminosMFPmod=modelsofarMFP,checkterms=allterms[index.terms$include])
                    varanova<-nueva.anova$anovaMFP
                    incluir.términos<-(1:length(attr(terms(modvar),"order")))[-
nueva.anova$terminos.MFP.repetidos]
                    } else {
                    varanova<-safeanova(modvar,type="marginal",checkterms=allterms[index.terms$include])[-1,]
                    incluir.términos<-(1:length(attr(terms(modvar),"order")))
                    }
    ########## Re-sort variables from varanova ##########
            var.rank.closed<-sort.list(varanova[,4], decreasing = TRUE,method="quick",na.last=NA)
            rank.sat.order<-(attr(terms(modvar),"order")[incluir.términos])[var.rank.closed]
            rank2.closed<-sort.list(rank.sat.order, decreasing = TRUE,method="radix")
            var.rank.closed<-var.rank.closed[rank2.closed]
            rank.sat.order<-rank.sat.order[rank2.closed]

            ##match allterms to rows in varanova:
```

```
            alltermscollapsed<-sapply(allterms,function (x)
paste(attr(terms(formula(paste("~",x))),"term.labels"),collapse="+"))

            var.rank.closed<-match(gsub(" ","",dimnames(varanova)[[1]]),gsub("
","",alltermscollapsed))[var.rank.closed]

            rank.out<-match(proxrank[1:variable],var.rank.closed,nomatch=0)
            rank.out<-switch(2-all(rank.out==0),1:length(var.rank.closed),-rank.out)

            proxrank<-c(proxrank[1:variable],var.rank.closed[rank.out])
            proxorder<-c(proxorder[1:variable],rank.sat.order[rank.out])


            print(as.name(paste("Variable", allterms[varranknext],
switch(index.terms$include[varranknext]+1,"excluded","incluided"))))

            ##History
            if (historial) rankprogesiv[[variable]]<-alltermsFIJO[proxrank]
            names(rankprogesiv)[variable]<-alltermsFIJO[varranknext]

            } #END variables





    ##################################################################################################
    ##################################################################################################
    #Convergence criteria:
    #All FP transformations are equal to the previous cycle
    #All included/excluded variables are the same
    convergencia<-
identical(MFP.p1[,index.terms$mfp],MFP.p0[,index.terms$mfp])*all(include.OLD==index.terms$include)
    if (convergencia) print(as.name(paste("Se hallo convergencia (ciclo",contador.ciclo,")")))

    ##History
    if (historial){
    Historico$ranking.progresivo[[contador.ciclo+1]]<-rankprogesiv
    Historico$incluidas<-rbind(Historico$incluidas,index.terms$include)
    Historico$MFP[[contador.ciclo+1]]<-MFP.p1[,index.terms$mfp]
    Historico$Anova[[contador.ciclo+1]]<-varanova
    }

    }#end ciclo (convergencia=0)


    ##############  FINAL MODEL  ###############
    #outputs from each cycle:
    #modvar
    #index.terms (particulary  $include)
    #allterms
    #MFP.p1
    #contador.ciclo

    modelo.final<-modvar
    inclusiones.final<-index.terms$include
    allterms.final<-allterms
    MFP.final<-MFP.p1


    ##History
    if (historial){
            names(Historico$ranking.progresivo)<-names(Historico$Anova)<-names(Historico$MFP)<-
dimnames(Historico$incluidas)[[1]]<-paste("cycle",0:contador.ciclo,sep=".")

            } ##history



    return(list(modelo.final=modvar
            ,transformaciones=MFP.final
            ,historia=Historico
            ,modelo.inicial=list(respuesta=as.name(response),nulo=paste(nuloterminos,collapse=" +
"),fijo=paste(fixterminos,collapse=" + "),confundentes=paste(confterminos,collapse=" +
"),FP=MFPon,FP.potencias=MFPpot)
            ,sinonimos=cbind.data.frame(allterms,alltermsFIJO)
            ,iteraciones=contador.ciclo
            ,call=caller
            ))
    } #END function .....      MFP.lme()
```
