# Supplementary material for: Hypercholesterolemia Is Associated with the Apolipoprotein C-III (APOC3) Genotype in Children Receiving HAART: An Eight-Year Retrospective Study
Source: PLoS One. 2012 Jul 25;7(7):e39678. doi: 10.1371/journal.pone.0039678 (PMC3405089; doi:10.1371/journal.pone.0039678)
Supplement: Discussion S1 — (PDF) [file pone.0039678.s010.pdf]

Supplementary Discussion

Statistical approach

We applied mixed factors models for the analysis of repeated determinations spaced along irregular time periods. Factors that could potentially modify the observed effect of APOC3 on lipid levels were included in the models, after a selection step through a backward algorithm. Models obtained and test run were pointed to the direction of the major robustness available to us, and showed consistency through parameters, assumptions or algorithm variations. Additionally, to avoid an over-interpretation due to an inflated type I error rate, a hierarchical approach was intended to weight statistically significant findings. Thus, definitive evidence of APOC3 association with lipid levels came from "global level" tests, while contrasts among genotypes were tested to answer post-hoc questions and to pose new hypothesis for future analysis. As a consequence, the resulting inferences tended to be rather conservative, probably in detriment of statistical power.

Despite maximum likelihood (ML) estimation for LMM and GLMM is a classical tool, other options exist for the analysis of correlated data that were particularly frequent in clinical studies, as is the case of generalized estimating equations (GEE)for marginal estimation[1]. The main advantage of GEE is that it does not assume a distribution of the response, and is robust to assumptions violations [2]. However, ML calculations for LMM/GLMM need a relaxed assumption on missing data, allowing a bias dependent on observed covariates and response. This is not the case of GEE, that assumes missing data to be conditionally independent of any unknown -*i.e.* not included in the model-individual characteristic [3]. Since the first determination available for each patient was not necessarily close to the date of HAART initiation and the final number of visits or the time extent sampled before the study end on December 2008 were unpredictable, missing data was of concern in the analysis of our cohort. In fact, patients with higher (mean) cholesterol levels in our sample presented a higher number of lipid determinations (p=0.003, Spearman's rank correlation test). This result could be explained with the differential management of patients with high cholesterol level who were more likely to have repeated measurements for a close monitoring of lipid abnormalities. This also meant that missingness was dependent on individuals' previously observed values, GEE assumptions were not realistic, and consequently, mixed models were more fit.

An step-wise procedure was applied for the selection of variables to be included in the final models for plasma lipid levels prediction and the most parsimonious functional form of continuous variables[4]. A concern with the application of this algorithm is type I error rate inflation, despite following a conservative modification for fractional polynomial procedures[5]. However, qualitative results from hierarchical tests were robust to variations on this algorithm or the analyzed variables, suggesting that there was not significant error inflation.

Our study differed from previous analyses of APOC3 influence on lipid levels in patients under HAART on the choice of the analytical approach, but also on the design of the study and parameters included. In particular, quantitative measures of the exposure to HAART were introduced, namely the time of exposure to the last

treatment regimen and the accumulated time on HAART. The inclusion was possible only through a long follow up for all patients, as a shorter study period, and thus a reduction of the number of determinations, would not have power enough to carry up a statistical test. Therefore, we realize that despite the inclusion of this quantitative measure is desirable, it might not be possible under other study settings. Besides the inclusion of time of exposure as a covariate, other parameters were taken into account, such as AIDS status –patients with AIDS had occasionally been excluded from previous studies[1]-, and menarche –only meaningful to children/adolescents lipid levels prediction-. Additional longitudinal variables (*i.e.* CD4+ T cells, viral load, BMI) that were previously adjusted were excluded from our final model. However, their inclusion showed no meaningful differences.

In conclusion, the choice of our statistical approach reflects both our data requirements and analysis criteria. Despite a different model parameterization hampers the comparison to previous work on APOC3, we chose the best parameters that suited our pediatric cohort. Furthermore, the inclusion of the time of exposure had an impact on the interpretation of our results, and it might likewise be of use in the analysis of other cohorts.

References for the Supplementary Discussion

1. Tarr PE, Taffe P, Bleiber G, Furrer H, Rotger M, et al. (2005) Modeling the influence of APOC3, APOE, and TNF polymorphisms on the risk of antiretroviral therapy-associated lipid disorders. J Infect Dis 191: 1419-1426.
2. Agresti A (2002) Categorical Data Analysis; Balding D, Bloomfield P, Cressie N, Fisher N, Johnston I et al., editors. Hoboken: John Wiley & Sons, Inc.
3. Hu FB, Goldberg J, Hedeker D, Flay BR, Pentz MA (1998) Comparison of population-averaged and subject-specific approaches for analyzing repeated binary outcomes. Am J Epidemiol 147: 694-703.
4. Sauerbrei W, Royston P (1999) Building multivariable prognostic and diagnostic models: Transformation of the predictors by using fractional polynomials. Journal of the Royal Statistical Society Series A 162: 71-94.
5. Ambler G, Royston P (2001) Fractional polynomial model selection procedures: investigation of type I error rate. Journal of statistical computation and simulation 69: 89-108.
