# Supplementary material for: Hypercholesterolemia Is Associated with the Apolipoprotein C-III (APOC3) Genotype in Children Receiving HAART: An Eight-Year Retrospective Study
Source: PLoS One. 2012 Jul 25;7(7):e39678. doi: 10.1371/journal.pone.0039678 (PMC3405089; doi:10.1371/journal.pone.0039678)
Supplement: Material and Methods S1 — (PDF) [file pone.0039678.s011.pdf]

# *Supplementary Material and Methods*

## *Statistical Analysis*

### *Longitudinal analysis of plasma lipid concentration*

Longitudinal data for total cholesterol (TC), triglycerides (TG), HDL-C and LDL-C plasma levels were analyzed separately by modeling the independent effects of genetic polymorphisms and additional covariates. The correlation between determinations from the same child was taken into account by fitting a Linear Mixed(-Effects) Model (LMM), a broadly used approach which includes "fixed effects" (population parameters associated with repeatable levels of experimental variables) and "random effects" (parameters associated with experimental units drawn at random, usually the sampled individuals as would be our case)[1]. Thus, the model for lipid levels $Y_{ij}$ measured for patient $i$ on the $j^{th}$ visit was (without interactions)

$$Y_{ij} = (\alpha + a_i) + \vec{\beta}\vec{T}_{ij} + \vec{\gamma}\vec{G}_i + \vec{\delta}\vec{Z}_{ij} + \varepsilon_{ij},$$

where the coefficients $\alpha$, $\vec{\beta}$, $\vec{\gamma}$ and $\vec{\delta}$ correspond respectively to the constant, time ($\vec{T}_{ij}$), genotype ($\vec{G}_i$) and other covariates ($\vec{Z}_{ij}$) fixed contribution. The model also assumed a common effect for all the lipid determinations within the same patient but with a random variation from patient to patient ("subject-specific" effect) and an error term $\varepsilon_{ij}$ with normal distribution and cero mean. This model conforms to the assumptions of a random-intercept model, a particular case of LMM. The best Box-Cox simple power transformation for the response variable in order to fulfill the error distribution normality assumption was previously assessed on a linear fixed-effects model. Maximum likelihood estimation of coefficients was carried out with the *nlme* package v3.1-96 [1] for CRAN R software v2.10.1[2]. There were several elements in the vectors of observations $\vec{T}_{ij}$, $\vec{G}_i$ and $\vec{Z}_{ij}$ at each determination that were potentially included in the final model. Hence, for $\vec{T}_{ij}$ the observed times since birth, to HAART initiation and of exposure to the last treatment regimen were assessed. Likewise, three loci in APOC3 were evaluated for $\vec{G}_i$ and additional covariates that were confounders or independent predictors of interest for $\vec{Z}_{ij}$, such as AIDS, patient age at first lipid determination, antiretroviral exposure before HAART, the set of drugs indicated at the time of each measure, plasma viral load and CD4+ T cell percentage (Supplementary Table S1). Additionally, interaction terms were alternatively included in the model to evaluate the fixed contribution of the time of exposure to each drug on the last treatment regimen, and timely variations on gene effect sizes. Box-Cox transformations were applied in order to stabilize variability and to achieve normality in the residuals of TC, HDL-C, LDL-C and TG. The most parsimonious functional form for each continuous scale predictor was chosen following first/second degree fractional polynomials algorithm with powers from the set [-1,-0.5,0 (log transformation), 0.5, 1 (no transformation),2][3]. Adequate functional form and selection of predictors for model growth were jointly approached with an iterative backward elimination algorithm –i.e." multiple fractional polynomials algorithm" -

modified from Sauerbrei and Royston[3,4]. Initial parameters for model growth are listed on Supplementary Table S1. The significance level to evaluate predictor exclusions was set to 0.05 for marginal likelihood ratio tests, whereas a higher significance of 0.2 was set for potential confounders (sex, menarche, AIDS, age, treatment) to ensure negligible residual confounding[5]. Significance for each predictor contribution on the final model was evaluated with hierarchical likelihood ratio tests under the null hypothesis of negligible effect with a Bonferroni corrected significance level of 0.003125. To find the best model for dependence among repeated measures from the same subject, correlation structures were evaluated with the Akaike information criteria, with the visual aid of empirical autocorrelation function graphs. However, consistent estimations for the tested predictors were found between every correlation structure. The distribution of likelihood ratio test statistics was confirmed with simulation as implemented on R package *nlme* v3.1-96.

## *Longitudinal analysis of hyperlipidemia events*

The regression for longitudinal data on binary outcomes was carried out under a Generalized Linear Mixed Model (GLMM) with a logit link function, that can be written:

$$\text{logit } \mathrm{P}\left(E_{ij} = 1 \mid patient = i\right) = \left(\alpha + a_i\right) + \vec{\beta}\vec{T}_{ij} + \vec{\gamma}\vec{G}_i + \vec{\delta}\vec{Z}_{ij},$$

where $E_{ij}$ may be 0 or 1, being 1 a hyperlipidemia event and $\mathrm{P}\left(E_{ij} = 1 \mid patient = i\right)$ is the probability of observing the event given the patient i, while the remaining terms are kept from LMM. Maximum likelihood estimation for odds ratios and 95% confidence intervals were obtained under this model with the R package *lme4* v0.999375-33[6]. No correlation structure for GLMM was implemented, as it was not an available feature on the current software version. Statistical significance for the contribution of each predictor to the risk of hyperlipidemia was evaluated under a hierarchical likelihood ratio test.

## *Linear mixed models building by multiple fractional polynomials algorithm*

Fractional polynomials (FP) algorithm is a method to select the most parsimonious transformation for continuous predictors, originally proposed by Royston [7], and with a broad application on epidemiology[8,9,10]. Previous applications of the FP algorithm were implemented on SAS, STATA and CRAN R for linear, logistic and Cox regression[11]. Despite FP application has been mainly applied for the analysis of data from cross-sectional studies, longitudinal models for repeated measures were also fitted following this algorithm[9]. Particularly, FP algorithm was applied to evaluate associations between genotype and lipid levels increase during HAART under longitudinal models with coefficients estimated with Generalized Estimating Equations (GEE)[12].

For the analysis carried out on this study, we developed an script on R to perform multiple FP algorithm when building the Linear Mixed Models (LMM), following a backward elimination stepwise regression, as proposed by Sauerbrei and Royston [4], with the algorithm corrections suggested by Ambler and Royston[3]. An additional modification was introduced to evaluate the inclusion of two-way interactions (second order terms). Briefly, this modification consists in simultaneously evaluating the same power transformations to the variable included in the first and second order terms. For further detail on the FP algorithm applied for the analysis of repeated measures of

lipid levels on children under HAART, we included as supporting information a flow diagram and the annotated CRAN R script (Supplemental Algorithm S1), implemented on version 2.10.1. The execution of this script needs *nlme* package installed.

## *References for Supplementary Material and Methods*


1. Pinheiro J, Bates D (2000) Mixed-effects models in S and S-PLUS. New York: Springer-Verlag.
2. (2010) R: A language and environment for statistical computing. Vienna: R Foundation for Statistical Computing. ISBN 3-900051-07-0 ISBN 3-900051-07-0.
3. Ambler G, Royston P (2001) Fractional polynomial model selection procedures: investigation of type I error rate. Journal of statistical computation and simulation 69: 89-108.
4. Sauerbrei W, Royston P (1999) Building multivariable prognostic and diagnostic models: Transformation of the predictors by using fractional polynomials. Journal of the Royal Statistical Society Series A 162: 71-94.
5. Royston P, Ambler G, Sauerbrei W (1999) The use of fractional polynomials to model continuous risk variables in epidemiology. International Journal of Epidemiology 28: 964-974.
6. Faraway JJ (2006) Extending the linear model with R : generalized linear, mixed effects and nonparametric regression models. Boca Raton: Chapman & Hall/CRC. ix, 301 p.
7. Royston P, Altman D (1994) Regression Using Fractional Polynomials of Continuous Covariates: Parsimonious Parametric Modelling (with discussion). Journal of the Royal Statistical Society Series C (Applied Statistics) 43: 429-467.
8. Royston P, Ambler G, Sauerbrei W (1999) The use of fractional polynomials to model continuous risk variables in epidemiology. Int J Epidemiol 28: 964-974.
9. Heine JJ, Land WH, Egan KM (2011) Statistical learning techniques applied to epidemiology: a simulated case-control comparison study with logistic regression. BMC Bioinformatics 12: 37.
10. Cui J, de Klerk N, Abramson M, Del Monaco A, Benke G, et al. (2008) Fractional Polynomials and Model Selection in Generalized Estimating Equations Analysis, With an Application to a Longitudinal Epidemiologic Study in Australia. American Journal of Epidemiology 169: 113-121.
11. Sauerbrei W, Meier-Hirmer C, Benner A, Royston P (2006) Multivariable regression model building by using fractional polynomials: Description of SAS, STATA and R programs. Computational Statistics & Data Analysis 50: 3464-3485.
12. Tarr PE, Taffe P, Bleiber G, Furrer H, Rotger M, et al. (2005) Modeling the influence of APOC3, APOE, and TNF polymorphisms on the risk of antiretroviral therapy-associated lipid disorders. J Infect Dis 191: 1419-1426.
