# Supplementary material for: Hypercholesterolemia Is Associated with the Apolipoprotein C-III (APOC3) Genotype in Children Receiving HAART: An Eight-Year Retrospective Study
Source: PLoS One. 2012 Jul 25;7(7):e39678. doi: 10.1371/journal.pone.0039678 (PMC3405089; doi:10.1371/journal.pone.0039678)
Supplement: Results S1 — (PDF) [file pone.0039678.s012.pdf]

Supplementary Results

Statistical Model validation

Since model growth algorithms result in the selection of only significant terms through sequential evaluation, they may cause the inflation of type I error rates by multiple testing. In order to avoid over-interpretation due to statistical significance inflation, the three alternative hypotheses depicted on Supplementary Figure S1 were evaluated for total cholesterol on initial parameters without selection by step-wise backward elimination. APOC3 association to a differential response along therapeutic regimens was additionally confirmed for models grown from initial parameters describing different drug combinations in more detail. Thus, model explicitly included genotype interaction with the patient use of RTV (aggregating regimens with RTV as booster or single PI), D4T, NNRTI and NFV. Global level results for genotypes showed consistency through corrections, still sustaining an APOC3 effect on cholesterol levels (Supplementary Table S5). Model diagnosis and tests simulation were also run and no major deviations to LMM assumptions were observed (Supplementary Figure S2). Simulations support an appropriate distribution of likelihood ratio test statistics, with a minimum p value variation -usually less than 1 order difference, in cases where a conservative correction was prudent-.

In order to analyze in more detail the functional form of the effect of exposure time on cholesterol levels and its interaction with APOC3 genotype, the final LMM was grown once again, but this time following a second degree fractional polynomials algorithm. The resulting model incorporated an additional regression term to explain the association with time of exposure. Thus, the difference between 3'UTR 3238 CG heterozygous and CC homozygous mean levels and increase after RTV boosted PI or D4T administration remained similar after model adjustments. However, some variation on the best functional form and strength of association of several factors was found (data not shown). Of interest, the two loci on IRE displayed shifts on the strength or even on the direction of the associations observed, the more drastic adjustments being observed for the interactions with the time of exposure. Despite these variations, global level significance tests for the hierarchical hypothesis posed were qualitatively the same (not shown). Projections for cholesterol levels on the complex model predict a sudden increase after regimen initiation –already seen on single time term model- followed by a gradual decrease along time (Supplementary Figure S3). Effect for different genotypes showed global curves shifts –conspicuous for 3'UTR 3238 locus- or slope changes –IRE -455 and IRE -482 loci.
